# Atomically Precise Enantiopure Bimetallic Janus Clusters

**DOI:** 10.1021/acscentsci.2c00754

**Published:** 2022-09-07

**Authors:** Yao Li, Qiu-Xu Zang, Xi-Yan Dong, Zhao-Yang Wang, Peng Luo, Xi-Ming Luo, Shuang-Quan Zang

**Affiliations:** † Henan Key Laboratory of Crystalline Molecular Functional Materials, Henan International Joint Laboratory of Tumor Theranostical Cluster Materials, Green Catalysis Center, and College of Chemistry, Zhengzhou University, Zhengzhou 450001, People’s Republic of China; ‡ College of Chemistry and Chemical Engineering, Henan Polytechnic University, Jiaozuo 454000, People’s Republic of China

## Abstract

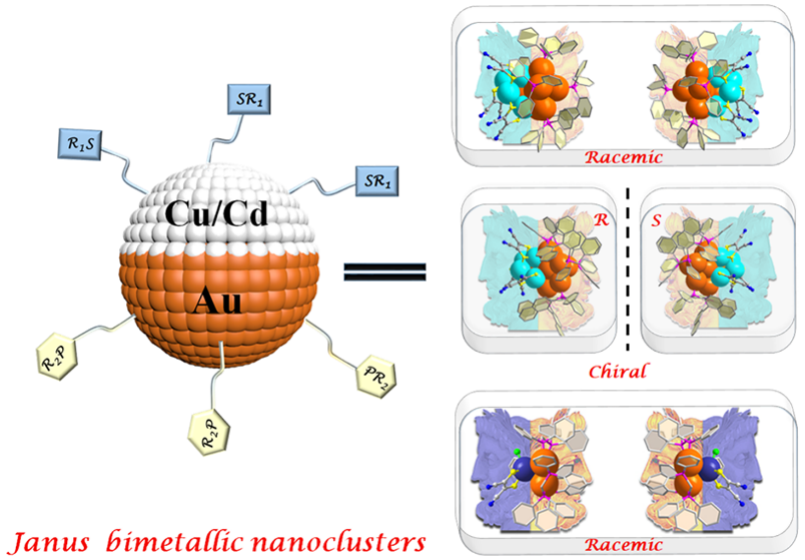

Asymmetric bimetallic Janus nanocrystals with a side-by-side interface have unique properties and important applications. However, understanding their fundamental issues, including their formation mechanism, interfacial linkage, and related properties, remains challenging, as does the preparation of enantiopure samples. Atomically precise Janus bimetal nanoclusters would unequivocally resolve these issues, yet they have not been realized. Here, based on Au and transition metals (Cu/Cd), and employing an S/P biligand strategy, we prepare and structurally resolve four Janus nanoclusters, including racemate 6e **Au**_**8**_**/Cu**_**4**_, 6e ***R***-/***S*-Au**_**8**_**/Cu**_**4**_ enantiomers, and 2e **racemateAu**_**3**_**/Cd**. Their interfacial linkage is unambiguously resolved at the atomic level, superatomic orbital splitting emerges, and unique molecule-like electronic transitions and chiroptical properties are present; more importantly, the dipolar distribution of bicomponents leads to a maximum dipole moment of up to 45 D, which drives the formation of 1D nanowires through self-assembly. This work provides a fundamental knowledge of intermetallic nanomaterials and an avenue for the synthesis of Janus nanoclusters.

## Abstract 2

Four bimetallic Janus clusters were reported, the dipolar distribution of bi-components leads to a maximum dipole moment up to 45 D, which drives the formation of 1D nanowires through self-assembly.

## Introduction

Structural and chemical heterogeneity^1^ in nanoarchitectures are highly attractive not only for the integration of multiple properties based on the individual components^2^ but also for new unique collective properties due to the possibility of rich electronic interactions between adjacent domains, which enhances their optics,^3^ plasmon,^4^ and catalysis properties.^5^ However, heterostructured intermetallic or bimetallic Janus systems with atomic ordering,^6^ but not a random arrangement or alloying,^3,7^ are dynamically unfavorable, requiring a delicate interplay balance between entropy and enthalpy; therefore, they are nearly unobtainable via direct mild bottom-up synthesis.^8^ Janus nanoarchitectures,^9^ named after the two-faced Roman god Janus, refer to nanoparticles with different surfaces or asymmetric structures. Bimetallic Janus nanocrystals^10^ are inherently asymmetric and should be a simple prototype for studying diverse intermetallic particles. Furthermore, homochiral Janus metallic nanoblocks can engender a type of new material because of their combination of physiochemical properties endowed by their nanoscale heterostructure and chiralty.^11,12^ Therefore, insight into the heterointerfaces of homochiral bimetallic Janus nanoparticles at the atomic scale becomes fundamentally important. However, preparing completely homogeneous homochiral Janus inorganic nanostructures and clearly resolving the interfacial structure, composition, and linkage between two hemisections remain remarkably challenging.

Metal nanoclusters,^13−15^ which are ultrasmall nanoparticles with sizes of 1–3 nm, demonstrate significant potential in elucidating fundamental nanoscience^16−19^ including asymmetry or chirality origins in metal nanoparticles.^20−22^ After incessant endeavors in the synthesis of atomically precise metal clusters, alloy^23−29^ and core–shell^30−33^ metal nanoclusters have exhibited great success in regard to their preparation and structural resolution. However, none have been reported for bimetallic Janus nanoclusters, not to mention enantiopure bimetallic Janus nanoclusters.

**Figure 1:**
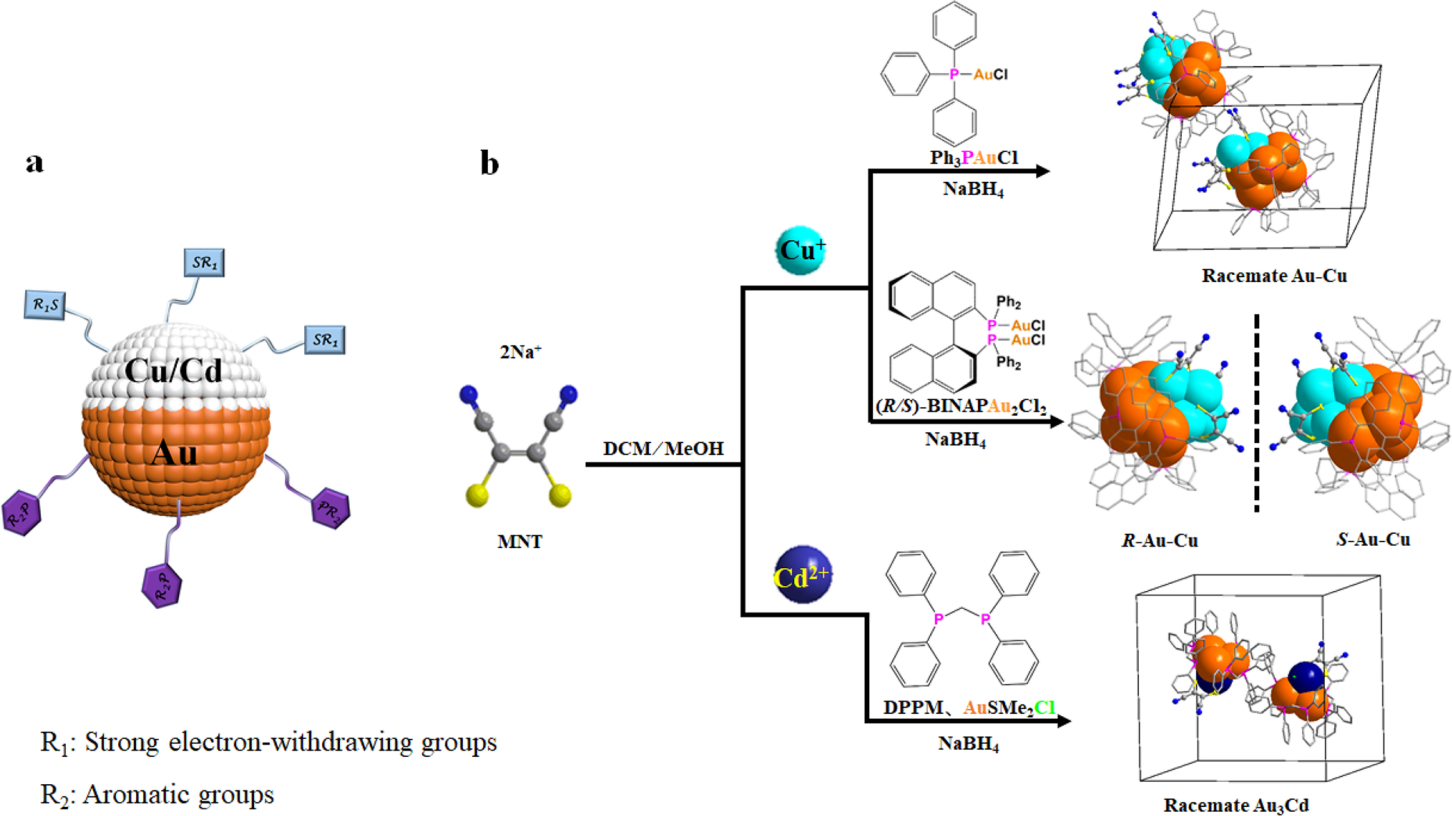
(a) Schematic of the bimetallic Janus structures. (b) Synthesis of **racemateAu-Cu**, and homochiral ***R-*/*S*-Au-Cu**, and racemate **Au**_**3**_**Cd**. Color legend: orange, Au; turquoise, Cu; pink, P; yellow, S; blue, N; gray, C.

**Figure 2:**
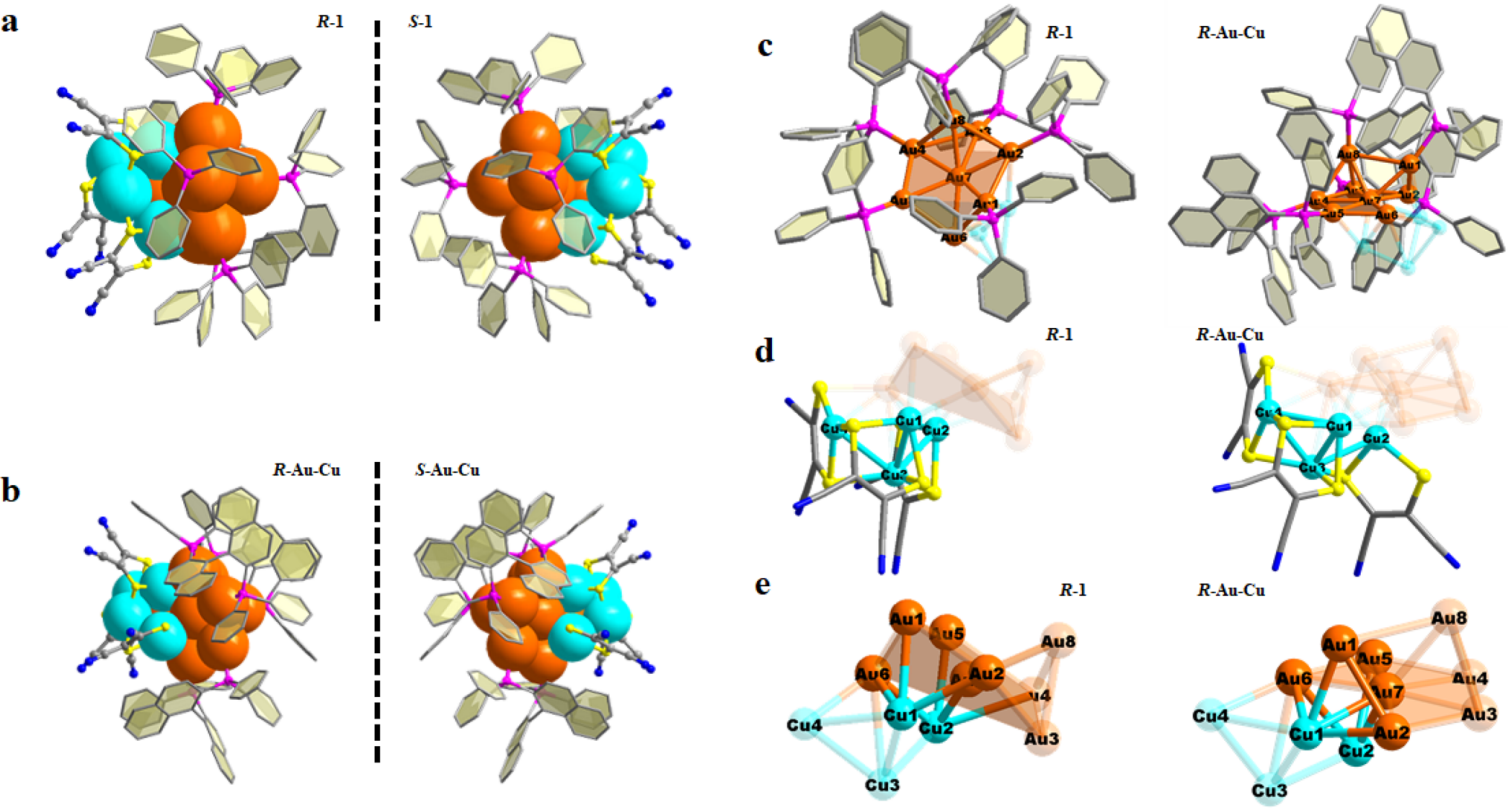
(a, b) Janus structures of *R*- and *S*-component clusters in racemic **Au-Cu** and ***R-***/***S*-Au-Cu**. The blue and orange Janus images embody the Cu-hemisphere and Au-hemisphere. (c–e) Comparison of the Au-hemisphere, Cu-hemisphere, and interdomain interface in the Au_8_Cu_4_ skeleton of ***R*****-1** and ***R*****-Au-Cu**. Color legend: orange, Au; turquoise, Cu; pink, P; yellow, S; blue, N; and gray, C. Hydrogen atoms are omitted for clarity.

Here we employ an S/P biligand strategy for preparing Janus nanoclusters based on Au and transition metals (Cu, Cd), in which the invariant bidentate S-based ligands di(sodiothio)maleonitrile (MNT^2–^) with strong electron-withdrawing C≡N groups have more affinity to Cu and Cd and the tunable monodentate/bidentate and achiral/chiral P-based ligands have more affinity to Au (Figure 1a and Figures S1–S5). As a result, four bimetallic Janus nanoclusters, 6e **racemateAu**_**8**_**/Cu**_**4**_, 6e ***R*/*S*-Au**_**8**_**/Cu**_**4**_ enantiomers, and 2e **racemateAu**_**3**_**/Cd** have been successfully prepared, the total structures of which are solved by using a single-crystal X-ray diffraction analysis (Figure 1b and Table S1). We demonstrate their asymmetric bipolar structures and unambiguous interfacial linkage (Figure 2). The 6e **racemate Au**_**8**_**/Cu**_**4**_ protected by achiral triphenylphosphine (TPP) and MNT^2–^ crystallizes in space group *Pn* with a pair of Au_8_(TPP)_6_/Cu_4_(MNT)_3_ clusters in one unit cell (Figure 1b), one of which is denoted as the *R* component (***R*****-1**) and the other as the *S* component (***S*****-1**). Further, using a pair of chiral diphosphine ligands *R*-/*S*-2,2′-bis(diphenylphosphino)-1,1′-binaphthyl) (BINAP) in place of achiral TPP on the Au hemisphere, a pair of enantiopure single crystals built from Au_8_(*R*-BINAP)_3_/Cu_4_(MNT)_3_ and Au_8_(*S*-BINAP)_3_/Cu_4_(MNT)_3_ (denoted ***R*-Au-Cu** and ***S*****-Au-Cu** ,respectively; Figure 1b) are obtained. The dipolar domain distribution of each Janus Au-Cu cluster results in the largest dipole moment of ∼45 D among the well-defined metal nanoclusters. When the transition metal Cd is used, the 2e racemate Janus Au_3_(DPPM)_3_/Cd(MNT)Cl (denoted **Au**_**3**_**Cd**; Figure 1b) is achieved, showing a dipole moment of ∼22 D. Interestingly, the chiral Au-Cu clusters are capable of spontaneously assembling into 1D nanoarrays through intercluster dipole interactions.

## Results and Discussion

### Synthesis of These Janus Nanoclusters

These Janus nanoclusters are synthesized by reducing a mixture of Au precursors (PPh_3_AuCl, *R*-/*S*-BINAPAu_2_Cl_2_, and AuSMe_2_Cl) and the corresponding transition-metal salts Cu(CH_3_CN)_4_PF_6_ and CdCl_2_ with NaBH_4_ at room temperature in the presence of Na_2_MNT (Figure 1b). **Au-Cu** clusters all crystallize as black-red single crystals, and the number of free valence electrons^34^ is calculated to be 6 (8Au + 4Cu(I)-3MNT^2–^ = 6). The **Au**_**3**_**Cd** cluster, crystallizing as red single crystals, has 2 free valence electrons (3Au + Cd(II)-MNT^2–^-Cl^–^ = 2). The phase purities, stability, compositions, valence, self-assembly, free electron number, and molecular formula of Janus clusters are further verified by PXRD, UV–vis absorption tracking, electrospray ionization mass spectrometry, energy dispersive spectrometry, X-ray photoelectron spectroscopy, TEM, silent electron paramagnetic resonance signals (Figures S6–S15), and density functional theory (DFT) (Figures S16–S24).

### X-ray Structures of **racemate Au-Cu** and ***R*-/*S*-Au-Cu**

Single-crystal structure analyses reveal that in **racemate Au-Cu** and ***R*-/*S*-Au-Cu** (Figure 2a,b) two hemispheres of each cluster are clearly parted: one part is either [**Au**_**8**_**(TPP)**_**6**_]^2+^ or [**Au**_**8**_**(***R***-BINAP)**_**3**_]^2+^ (abbreviated as Au-hemisphere) and the other [**Cu**_**4**_**(MNT)**_**3**_]^2–^ (abbreviated as Cu-hemisphere) (Figure 2c,d). Taking an example of ***R*****-1** in **racemate Au-Cu**, (Figure 2c–e), in the Au-hemisphere, six Au atoms form a twisted hexagon, five of which are ligated with five TPP ligands, with a naked seventh Au atom in the center of the hexagon. The eighth Au atom protrudes outward and bonds with a TPP (Figure 2c and Figure S1). The Au–Au separations^35,36^ are in the range of 2.66–3.04 Å. In Cu-hemisphere (Figure 2d), four Cu atoms are weakly bonded by Cu–Cu contacts (2.75–2.90 Å) and ligated by three MNT^2–^ ligands. Each MNT^2–^ chelates a Cu atom, and three MNT^2–^ ligands together hold the fourth Cu atom by using a S atom. The bias to Cu of MNT^2–^ and that of P-based ligands (TPP and BINAP) to Au atoms are clearly evidenced. In the interface between the Au/Cu domain, rich Au–Cu bonding (2.57–2.77 Å) increases the rigidity of the cluster skeleton (Figure 2e and Table S2), which provides the possibility for communications between two hemispheres.

This distinct biphasic model, P-ligated Au and S-ligated Cu, in the chiral cluster component in **racemate Au-Cu** crystals inspired us to engineer chiral ligands for homochiral Janus nanoclusters. We used an available enantiomeric pair of diphosphine ligands (*R*-/*S*-BINAP) with axial chirality to replace the achiral TPP in the Au-hemisphere (Figure 2a,b). Thus, enantiopure ***R***-/***S*-Au**_**8**_**(***R***-BINAP)**_**3**_**/Cu**_**4**_**(MNT)**_**3**_ were prepared, which basically retain a Au_8_/Cu_4_ skeleton similar to that in **racemateAu-Cu**, yet with different metallic bond angles and lengths (Tables S2 and S3). From ***R*****-1** to ***R*****-Au-Cu**, we find that the order of the metal skeleton increases (Figure 2e). The heptamer Cu_1_Au_6_ is pulled to a more regular Au-centered hexagonal plane because of the tight chelation of the diphosphine ligand (Figure S2). This deformation of the Au_8_Cu_4_ skeleton leads to a change in the interface between the Au-hemisphere and Cu-hemisphere (Figure 2e). Compared to **racemate Au-Cu**, which is more soluble in polar DMF and DMAc solvents, the solubility of ***R-***/***S*-Au-Cu** crystal changes considerably and is easily soluble in organic solvents (CH_2_Cl_2_, CH_3_Cl).

**Figure 3:**
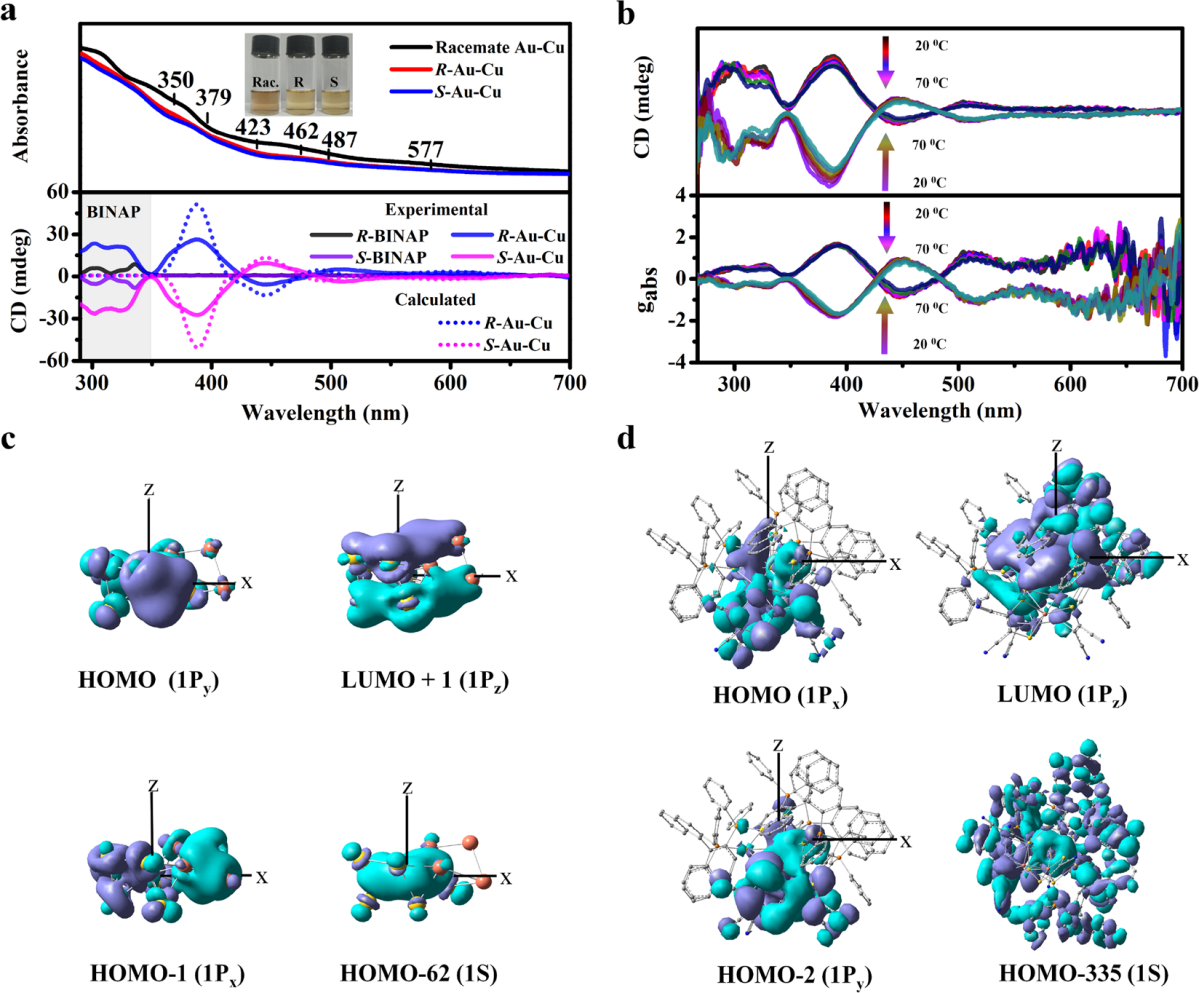
(a) UV–vis spectra of the **racemateAu-Cu** and ***R-***/***S*-Au-Cu** solution (5 × 10^–4^ M in DMF) and experimental CD spectra of the *R*-/*S*-BINAP ligand and ***R-***/***S*-Au-Cu** (5 × 10^–4^ M in dichloromethane). Calculated CD spectra of ***R-***/***S*-Au-Cu** have a 16 nm red shift. (b) Temperature-dependent absorbance spectra and *g*_abs_. (c) Superatomic orbitals of naked [Au_8_Cu_4_]^6+^ in ***R*****-Au-Cu**. (d) Superatomic orbitals in the complete ***R*****-Au-Cu** cluster.

The UV–vis absorption spectra of both **racemate Au-Cu** and ***R-***/***S***-**Au-Cu** demonstrate molecule-like absorbance peaks (Figure 3a). Additionally, the P-based ligands obviously affect the electronic transitions, showing a shift in the range of 400–500 nm, where **racemate Au-Cu** has a broad peak at 462 nm and ***R-***/***S*-Au-Cu** has two absorbance peaks at 423 and 487 nm. These differences in the electronic transitions in the visible region are ascribed to the deformation of the metal skeleton of Au_8_Cu_4_, which results in the slightly different electronic structures (Figures S16 and S17). The calculated UV/vis spectra are basically consistent with the experimental results except for a small systematic red shift (Figures S18 and S19 and Tables S4 and S5). The stability of the **racemate Au-Cu** and ***R*****-Au-Cu** NCs in solution was also proved by time-dependent UV–vis absorption (Figure S7). For the ***R*****-Au-Cu** NCs, the large dipoles also led to strong interactions between NCs and solvent molecules in solution. Here, we have measured the solvent-dependent UV–vis spectra of the ***R*****-Au-Cu** NCs by using dichloromethane (CH_2_Cl_2_), chloroform (CHCl_3_), *N*,*N*-dimethylformamide (DMF), and dimethyl sulfoxide (DMSO) (Figure S8). The absorption peaks of ***R*****-Au-Cu** NCs show distinct shifts (∼80 meV) depending on the polarity of the different solvents, further showing the large dipole of NCs at the ground state.

The CD spectra of ***R-***/***S*-Au-Cu** exhibit nearly perfect mirror-image signals from 260 to 660 nm, in which the signals before 350 nm arise from the chiral BINAP ligands. Cotton effects involving the transitions related to valence electrons appear at 446, 507, and 620 nm, which are reproduced in the calculated results (Figure 3a). The anisotropic factor (*g*_abs_) is calculated for the whole spectrum with an anisotropic factor of ∼1.5 × 10^–3^, which is comparable to those reported in the literature,^37^ and it is nearly independent of temperature (Figure 3b). However, the absorbance intensity slightly decreases as the temperature is increased from 20 to 70 °C, indicating the rigid chirality.

### Density Functional Theory (DFT) Calculations and Electronic Structure

To understand what effects the ligand has on the electronic structures, we performed DFT calculations on four models of the Au-Cu Janus systems: i.e., two ligand-free metal skeletons of [Au_8_Cu_4_]^6+^ in ***R*****-1** and ***R**-***Au-Cu** and whole clusters with complete ligands (Figure 3c,d and Figures S20 and S21). For the [Au_8_Cu_4_]^6+^ skeleton in ***R*****-Au-Cu** (Figure 3c), the superatomic 1s orbital is found in HOMO-62, and the 1p superatomic orbitals are located in the HOMO-1 (1p_*x*_), HOMO (1p_*y*_), and LUMO+1 (1p_*z*_) orbitals. This model indicates that Au-Cu bidomains can fuse together through the interface for delocalization over the bimetallic skeleton due to the presence of Au–Cu bonds (Table S2); notably, this can occur despite the Au-Cu lattice mismatch of up to 12%^38^ and the large difference in atomic radius (Au 1.44 Å vs Cu 1.28 Å). Au–Cu interactions may play more critical roles in the binary metal nanoclusters, and the lattice adaptability from the protecting ligands induces asymmetric growth.

When ligands ligate [Au_8_Cu_4_]^6+^ (Figure 3d), the typical superatomic orbitals of clusters become complicated by the hybridization of the ligands, and the electron distribution changes considerably. In the HOMO-335 orbital, we find the characteristics of the s orbital, while the HOMO orbital exhibits the characteristics of p_*x*_, the HOMO-2 orbital exhibits the characteristics of p_*y*_, and the LUMO orbital exhibits the characteristics of p_*z*_ in the Au-hemisphere. In addition, the electron clouds also pulled toward the side of the Cu-MNT, indicating that the two HOMOs have considerable contributions from MNT^2–^, which engenders the separated states of charges within the cluster. For ***R*****-Au-Cu**, the HOMO and HOMO-2 and the LUMO are localized in the Au-hemisphere and MNT-ligated Cu-hemisphere with a small contribution of P-based ligands (BINAPs). A similar trend is applicable for ***R*****-1** in **racemate Au-Cu** (Figures S20 and S21). In addition, through the chiral BINAP instead of the achiral TPP on the surface of the Au-hemisphere, we not only achieve enantiomeric separation of racemate Janus clusters but also modulate the electronic structure by finely adjusting the geometric architecture of the Au-Cu skeleton. Although Au-Cu alloying^39,40^ and core–shell Au@Cu^41^ are predominant among the previously reported libraries of polyelemental nanoparticles and nanoclusters, this finding is the first to exemplify the possibilities of a homochiral biphasic Au-Cu system, which is also the smallest enantiopure Janus bimetallic structure with a well-defined composition and geometry.

**Figure 4:**
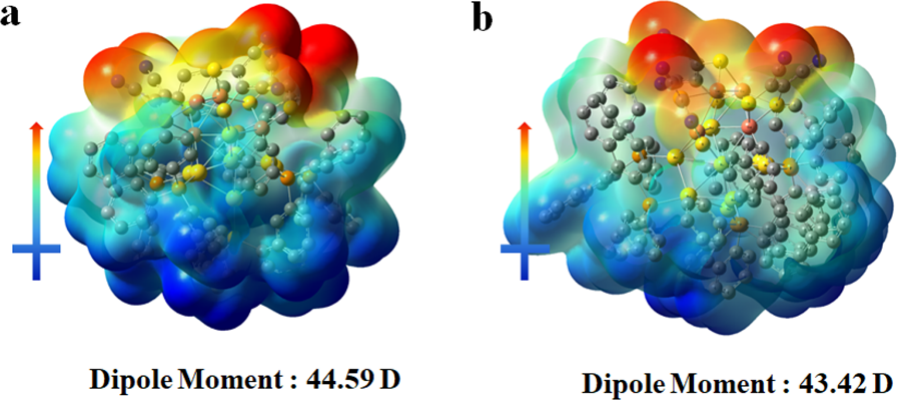
(a, b) Direction and value of the dipole moment of the ***R*****-1** and ***R*****-Au-Cu** clusters. Red represents the electron-rich region, and blue denotes the electron-poor region.

The calculations of the dipole moments demonstrate that the μ value of ***R*****-1** is ∼44.6 D and is ∼43.4 D for ***R*****-Au-Cu** (Figure 4), which to the best of our knowledge is the largest value among reported well-defined metal nanoclusters.^42^ We temporarily presume that the unique dipolar Janus structure and the distributed six valence electrons could be responsible for the large polarity of these clusters. In racemate **Au-Cu**, the *R*- and *S*-cluster components are alternatingly arranged; in crystalline ***R*****-Au-Cu**, chiral Janus clusters are located along the 2_1_ screw axis with the orientation of the dipole moment aligned along 2_1_, basically forming a “head-to-tail” alignment with significant dipole–dipole interactions throughout the crystal (Figure S3).

### Self-Assembly of ***R*-Au-Cu** Nanoclusters

**Figure 5:**
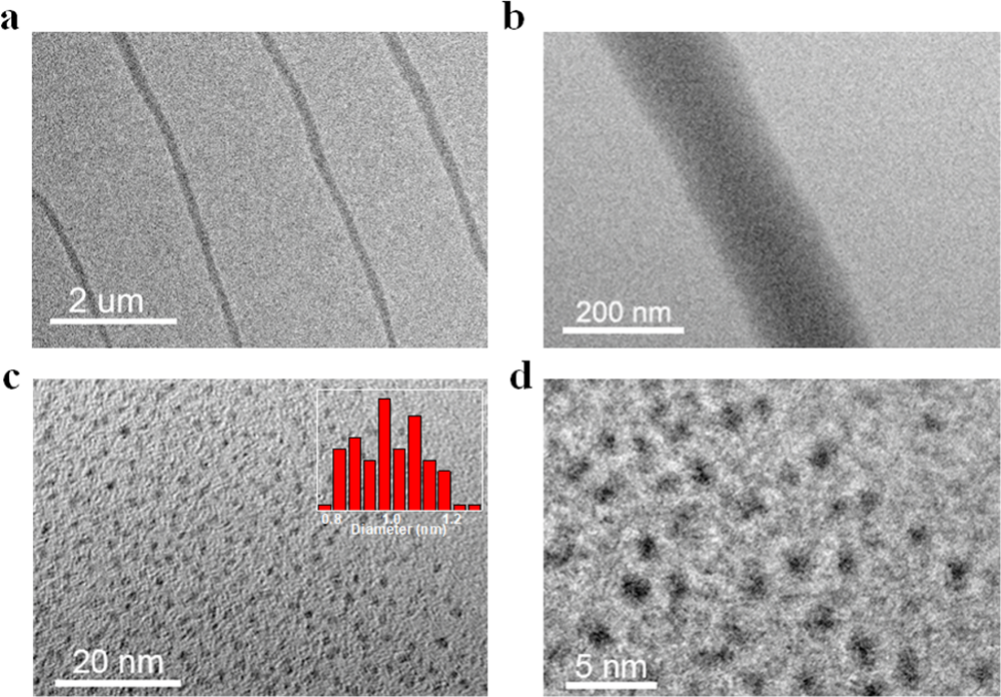
(a) TEM image of nanowires made from ***R*****-Au-Cu** at the micrometer scale. (b–d) TEM images of the observed nanowires at different magnifications.

From the “head-to-tail” arrangement and the characteristics of the large dipole moment of the ***R*****-Au-Cu** nanoclusters, whether they can assemble into superstructures aroused our interest.^43^ Fortunately, when we dropcasted the ***R*****-Au-Cu** clusters solution onto the TEM grid, the clusters spontaneously assembled into 1D micrometer-scale nanowires at room temperature (Figure 5a). To further analyze the reason for the self-assembly of ***R*****-Au-Cu** nanoclusters into 1D nanowires, we observed the nanowires at different magnifications (Figure 5b,d). We counted the size of the ***R*****-Au-Cu** nanoclusters at the 20 nm scale and found that the size was mainly concentrated in the range 0.82–1.17 nm, which was close to the size of the cluster itself, indicating that the cluster was a monodisperse state. Therefore, the ***R*****-Au-Cu** cluster can be considered the foundational-level (primary structure) building blocks of the nanowires. In addition, the distance between the two clusters was distributed in the range 1.8–2.5 nm, which is close to the cluster spacing of the clusters in the solid state, indicating the prescence of dipole interactions in the nanowires. For the ***R*****-Au-Cu** cluster, the dipole moment was as high as μ = 43.42 D; the energy of the dipole attraction between nanoclusters can be calculated with the classical formula for aligned dipoles *E* = −μ^2^(3 cos θ_1_ cos θ_2_ – cos θ_12_)/4πε_0_*r*^3^ (ε_0_ = 8.85 × 10^–12^ C^2^ J^–1^ m^–1^),^44^ which can be as high as 37.54 kJ/mol in the solid state (Figure S4). van der Waals forces between anisotropic nanoclusters cores can contribute to the unidirectional aggregation of the clusters. However, they are too weak to stabilize nanoparticle superstructures under ambient conditions. Therefore, the forces capable of producing nanowires of nanoclusters were considered to be the inherent bipolar phase and intercluster dipole interactions.^44−46^

### Janus **Au_3_Cd** Clusters

The asymmetric Janus **Au**_**3**_**Cd** cluster crystallizes in the form of a racemate (Figure 1b). Each **Au**_**3**_**Cd** cluster forms a regular tetrahedron (Figure S5), where three Au molecules ligated by three dppm ligands surround a triangle with a Au–Au side of 2.79–2.84 Å. The Au–Cd bond lengths range from 2.85 to 2.90 Å, agreeing with the reported value.^47,48^ The single Cd atom is chelated with a MNT ligand and coordinated with a Cl^–^ ion. To the best of our knowledge, **Au**_**3**_**Cd** is the smallest Janus superatomic cluster. Its structure and characterizations are given in the Supporting Information.

The UV–vis absorption spectrum of **Au**_**3**_**Cd** in a DCM solution exhibits three prominent peaks at 253, 318, and 500 nm, which were well reproduced in the calculated UV/vis spectra from time-dependent density functional theory (Figure S22 and Table S6). The stability of **Au**_**3**_**Cd** in solution was also proved by time-dependent UV–vis absorption (Figure S7). **Au**_**3**_**Cd** has two valence electrons according to the superatom model.^34^ In the HOMO orbital, we found the characteristic of the s orbital, and the electron cloud is delocalized in the Au_3_Cd framework (Figure S23). In addition, the electron cloud of the HOMO is pulled to the side of Cd-MNT, indicating that the MNT ligand has an important effect on the HOMO of **Au**_**3**_**Cd**.

The dipole moment of a Janus **Au**_**3**_**Cd** cluster is calculated to be ∼21.85 D (Figure S24), which is larger than those recently reported for Au_19_Ag_3_Cd_1_(SAdm)_15_Br, Au_22_Cd_1_(SAdm)_15_Br, and Au_19_Ag_4_(SAdm)_15_.^42^ The results of the **Au**_**3**_**Cd** cluster also demonstrate the effectiveness of using a mixed ligand strategy. Phosphorus-based ligands with more Au affinity and bidentate sulfur-based ligands (MNT^2–^) with more Cu (Cd) affinity result in the Au and Cu (Cd) presenting a Janus distribution. We propose that the arrangement of ligands with large differences in electronegativity and heterometals in our Janus structure is the main reason for the large dipole moment.

### Photocurrent Response Properties

Because these clusters have large dipole moments along different directions in the single-crystal structure and excellent optical absorption properties,^49,50^ we first tested the photocurrent response properties of **racemate Au-Cu**, ***R*****-Au-Cu**, and **Au**_**3**_**Cd** (Figure S25) using powder samples. The results show that they have good photogenerated electron/hole pair generation and separation efficiencies. The photocurrent density was maintained after multiple cycles, indicating that the response had good reproducibility. Then, we test photoelectric response by using the larger single crystals of ***R*****-Au-Cu**, which is easier to determine (Figure S26). When the laser is switched on/off, the photocurrent density reversibly increases and decreases correspondingly, and the photocurrent densities along the *c* axis for ***R*****-Au-Cu** single-crystal devices is ∼2.9 times higher than those along the *b* axis (Figure S27). We tentatively ascribed the anisotropic photoelectronic response to the different intercluster spacing, 1.61 nm along the *c* axis and 2.68 nm along the *b* axis, and the distinct dipole interactions.

## Conclusion

In conclusion, we prepared the smallest enantiopure Janus bimetallic cluster by using mixed ligands with different metallic affinities and electronegativities of the substituents; moreover, the finest asymmetric structure was demonstrated. The ligands severely changed the superatomic orbitals that were originally located on the bimetal cores and redistributed the valence electrons, enabling the bipolar domain in clusters and hence the largest dipole moments among metal nanoclusters, which drives these nanoclusters to self-assemble into 1D nanowires, providing a new possible way to well-ordered superstructures. This work on prototypical well-defined chiral Janus systems offers a direction in which to look for the design of chiral nanoclusters (nanoparticles) and provides possible applications in the fields of optical–electronic devices and catalysis and single-cluster nanomotors driven by magnetic fields, electrical fields, and/or local chemical energy.

## Supporting Information Available

X-ray data for Au-Cu clusters (CCDC numbers) **racemate Au-Cu** (2151533), **racemate Au-Cu** (2151533), ***R*****-Au-Cu** (2151535), and ***S*****-Au-Cu** (2151534), X-ray data for the Au-Cd cluster (CCDC numbers) **Au**_**3**_**Cd** (2151532), and additional experimental details, methods, materials, characterizations, theoretical calculations, photoelectric responses, and crystallographic data (PDF)
Crystallographic data for racemate Au-Cu (CIF)
Crystallographic data for *R*-Au-Cu (CIF)
Crystallographic data for *S*-Au-Cu (CIF)
Crystallographic data for Au_3_Cd (CIF)
CheckCIF/PLATON report for racemate Au-Cu (PDF)
CheckCIF/PLATON report *R*-Au-Cu (PDF)
CheckCIF/PLATON report for *S*-Au-Cu (PDF)
CheckCIF/PLATON report for Au_3_Cd (PDF)

## Supplementary Material

oc2c00754_si_001.pdf

oc2c00754_si_002.cif

oc2c00754_si_003.cif

oc2c00754_si_004.cif

oc2c00754_si_005.cif
