# Supplementary material for: Atomically Precise Enantiopure Bimetallic Janus Clusters
Source: ACS Cent Sci. 2022 Sep 7;8(9):1258–64. doi: 10.1021/acscentsci.2c00754 (PMC9523771; doi:10.1021/acscentsci.2c00754)
Supplement: Supplementary file 1 — oc2c00754_si_001.pdf [file oc2c00754_si_001.pdf]

Supporting Information

Atomically Precise Enantiopure Bimetallic Janus-Superatomic clusters

Yao Li,[1] Qiu-Xu Zang,[1] Xi-Yan Dong,[1,2] Zhao-Yang Wang,[1] Peng Luo,[1,2] Xi-Ming Luo,[1] and Shuang-Quan Zang[1,*]

[1]Henan Key Laboratory of Crystalline Molecular Functional Materials, Henan International Joint Laboratory of Tumor Theranostical Cluster Materials, Green Catalysis Center, and College of Chemistry, Zhengzhou University, Zhengzhou 450001, China.

[2]College of Chemistry and Chemical Engineering, Henan Polytechnic University, Jiaozuo 454000, China.

*E-mail: zangsqzg@zzu.edu.cn

Section S1: Materials and Methods

Materials and Reagents.

Sodium borohydride ($NaBH_4$, 98%), triphenylphosphine (PPh_3, 99%), tetrakis(acetonitrile)copper(I) hexa-fluorophosph, (S)-(-)-2,2'-bis(diphenylphosphino)-1,1'-binaphthyl, (R)-(+)-2,2-bis(diphenylphosphino)-1,1-binaphthyl, gold (III) chloride hydrate were purchased from Energy Chemical. Na_2MNT was purchased from Jiangsu Beida Pharmaceutical Technology Co., Ltd. Dichloromethane (CH_2Cl_2, A.R.), and methanol (CH_3OH, A.R.) were purchased from Sinopharm Chemical Reagent Co. Ltd. (Shanghai, China). All reagents were used as received without further purification.

Instrumentation.

UV-Vis absorption spectra were recorded using a Hitachi UH4150 UV-Visible spectrophotometer in the range 200-800 nm. Circular dichroism (CD) spectra were recorded by a Chirascan V100 spectropolarimeter in dichloromethane (DCM) solution. X-ray photoelectron spectroscopy (XPS) was recorded using ThermoFisher ESCALAB 250XI with Al Kα as excitation source. Binding energy were calibrated by C 1s at 284.8 eV. HRESI-TOF-MS spectrum were collected on a Sciex X500R Q-TOF spectrometer. EDS measurement was carried out using Zeiss Sigma 500. High-resolution transmissionelectron microscopy (TEM) studies were performed on a Tecnai G2 F20S. Powder X-ray diffraction data was acquired at room temperature in air using a Rigaku MiniFlex diffractometer (Cu-Kα; λ = 1.54178 Å; 2θ range of 3-50°). Anisotropic photocurrent was measured by two-probe method with a semiconductor parameter analyzer (B1500A, Keysight), and the electrodes device is in a probe station (CGO-4, Cindbest). A 450 nm light with a power density of 1 mW/cm^2 was used as the light source for the optical switch characteristics measurements.

Single-Crystal X-ray Diffraction Analysis.

Single-crystal X-ray diffraction measurements of the **racemate Au-Cu**, ***R/S*-Au-Cu** and **Au_3Cd** complexes were performed with a Rigaku XtaLAB Pro diffractometer. Data collection and reduction were performed using the program CrysAlisPro.[1] All the structures were solved by intrinsic phasing methods (*SHELXT-2015*)[2] and refined by full-matrix least squares on F^2 using Olex2,[3] which utilizes the *SHELXL-2015* module.[4] All nonhydogen atoms were placed based on electron density and refined with anisotropic thermal parameters, and hydrogen atoms were placed in calculated positions with idealized geometries and assigned fixed isotropic displacement parameters.

For **racemate Au-Cu**, appropriate restraints (DFIX, DELU, ISOR) were applied to the geometry, and the atomic displacement parameters of the atoms in the clusters were determined. Due to the disordered PPh_3 ligands, DFIX restraints were applied to keep the P–C bond length (1.85 Å) in a reasonable range. DELU and ISOR restraints were used for some C, P and Cl atoms with large thermal motion. The disordered solvent molecules in the structure could not be well identified, so the 'Solvent Mask' was adopted to remove them during structural refinement.

For ***R*-Au-Cu**, appropriate restraints (DFIX, SIMU, ISOR) were applied to the geometry, and the atomic displacement parameters of the atoms in the clusters were determined. Due to the less than ideal resolution, DFIX restraints were applied to keep the C–C bond length (1.39 Å) of the benzene ring in a reasonable range. SIMU and ISOR restraints were used for some C, N and Cl atoms from some organic ligands with large thermal motion. The disordered solvent molecules in the structure could not be well identified, so the 'Solvent Mask' was adopted to remove them during structural refinement.

For ***S*-Au-Cu**, appropriate restraints (DFIX, DELU, ISOR) were applied to the geometry, and the atomic displacement parameters of the atoms in the clusters were determined. Due to the less than ideal resolution, DFIX restraints were applied to keep the C–C bond length (1.39 Å) of the benzene ring in a reasonable range. DELU and ISOR restraints were used for some C, N and Cl atoms from some organic ligands with large thermal motion. The disordered solvent molecules in the structure could not be well identified, so the 'Solvent Mask' was adopted to remove them in structural refinement.

For **Au_3Cd**, appropriate restraints (DFIX, FLAT, DELU, ISOR) were applied to the geometry, and the atomic displacement parameters of the atoms in the clusters were determined. Due to the less than ideal resolution, DFIX restraints were applied to keep the C–C bond length (1.39 Å) of the benzene ring in a reasonable range. FLAT restraints were applied to maintain the planarity of the benzene ring. DELU and

ISOR restraints were used for some C, N and Cl atoms from some organic ligands with large thermal motion.

Section S2: Synthesis

Synthesis of single crystals of racemate of $Cu_4Au_8(TPP)_6(MNT)_3$.
A mixture of Na_2MNT (0.02 mmol), $AuPPh_3Cl$ (0.02 mmol), and $Cu(CH_3CN)_4PF_6$ (0.018 mmol) was added to 2 mL of methanol and 2 mL of dichloromethane and vigorously stirred with a magnetic stirring bar. After 20 min of stirring, 3 mg of $NaBH_4$ was quickly added with vigorous stirring. The reaction was performed for 24 hours at room temperature. Black–red block crystals in a yield of 20% were obtained after several days of slow solvent evaporation. Elemental analysis (%) for evacuated **$Cu_4Au_8(TPP)_6(MNT)_3$**: calcd C: 37.69, H: 2.37, N: 2.20, S: 5.03; found C: 37.53, H: 2.31, N: 2.16, S: 4.92.

Synthesis of homochiral single crystals of *R/S*-$Cu_4Au_8(BINAP)_3(MNT)_3$.
Similar to the synthesis of $Cu_4Au_8(TPP)_6(MNT)_3$, with the exception of the use of 0.01 mmol of (*R*-$BINAPAu_2Cl_2$)/(*S*-$BINAPAu_2Cl_2$) in place of 0.02 mmol of $AuPPh_3Cl$. Black–red block crystals in a yield of 5% were obtained after several days of slow solvent evaporation. Elemental analysis (%) for evacuated ***R*-$Cu_4Au_8(BINAP)_3(MNT)_3$**: calcd C: 42.00, H: 2.35, N: 2.04, S: 4.67; found C: 41.73, H: 2.27, N: 1.75, S: 4.13.

Synthesis of single crystals of $Au_3Cd(DPPM)_3(MNT)Cl$.
A mixture of Na_2MNT (0.02 mmol), $CdCl_2$ (0.03 mmol), $AuSMe_2Cl$ (0.02 mmol) and DPPM (0.02 mmol) was added to 2 mL of methanol and 2 mL of dichloromethane and vigorously stirred with a magnetic stirring bar. Then, 3 mg of $NaBH_4$ was quickly added with vigorous stirring. The reaction was aged for 12 hours at room temperature. Red block crystals in a yield of 25% were obtained after several days of slow solvent evaporation. Elemental analysis (%) for evacuated **$Au_3Cd(DPPM)_3(MNT)Cl$.**: calcd C: 46.69, H: 3.27, N: 1.38, S: 3.16; found C: 46.77, H: 3.21, N: 1.22, S: 3.11.

Section S3 Structures.

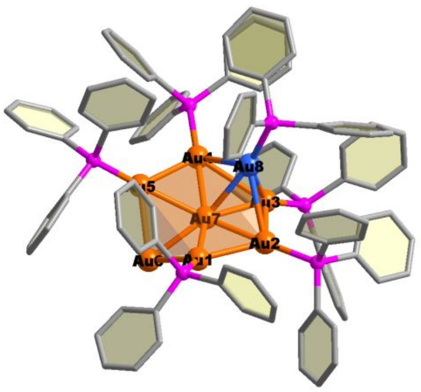

Figure S1. The Au-hemiphere in **racemate Au-Cu**, Au8 is highlighted in light blue. Au1 to Au6 form a twisted hexagon, and Au7 is located in the center of the hexagon.

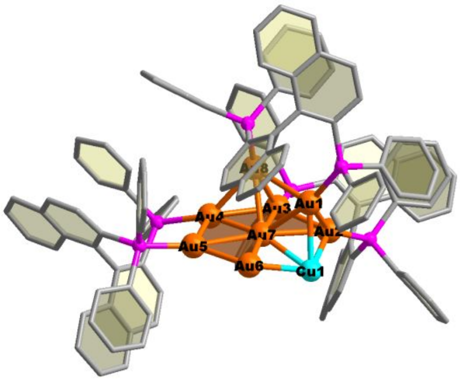

Figure S2. The Cu_1Au_8 in ***R*-Au-Cu**. Compared to **Racemate Au-Cu**, the three bidentate phosphine ligands chlate Au1 and Au8, Au2 and Au3, Au4 and Au5, respectively.

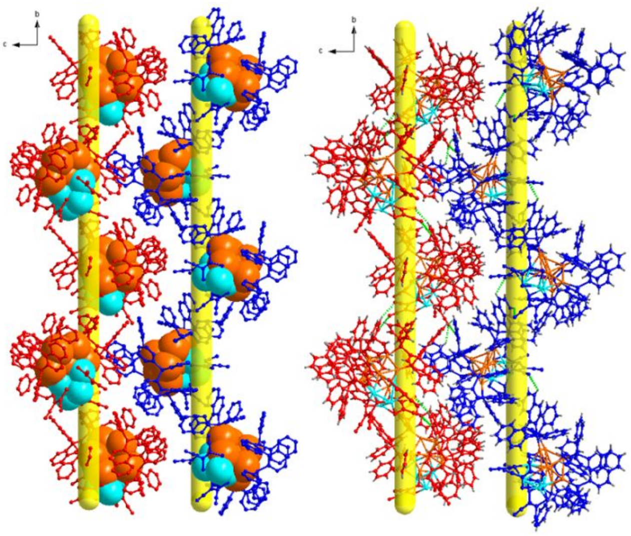

Figure S3. Arrangement of the ***R*-Au-Cu** cluster along the a-axis. The ***R*-Au-Cu** cluster are located along 2_1 screw axis with the orientation of dipole moment aligned 2_1, and forming "head-to-tail" alignment. It was found that C-H ··N hydrogen bonds (2.45 - 2.88 Å) between MNT and naphthalene groups of BINAP ligands.

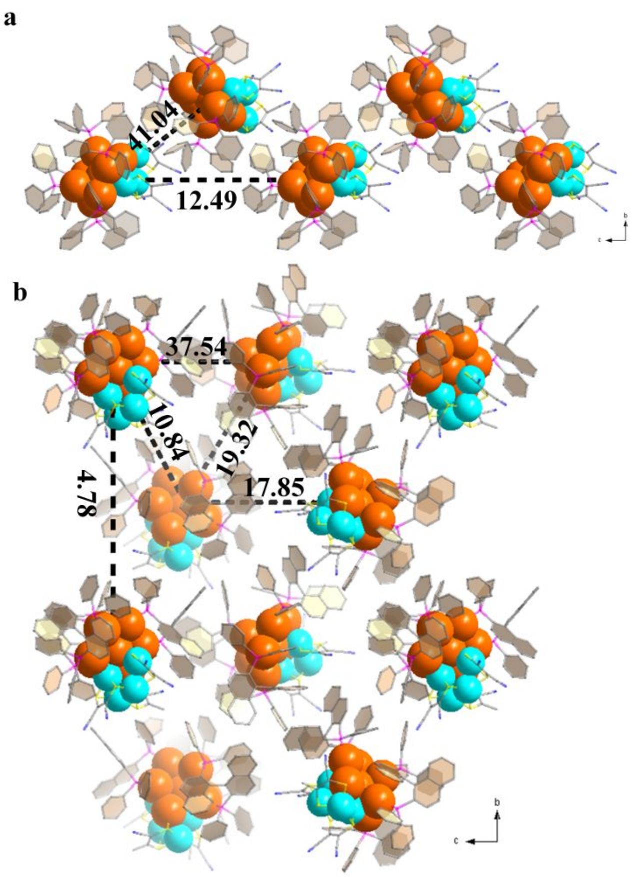

Figure S4. NC dipole alignment in the crystal states of **racemate Au-Cu** (a) and ***R*-Au-Cu** (b) the energy of attraction interactions between two NC dipoles are indicated in the unit of kJ/mol. Color legend: orange sphere, Au; turquoise sphere, Cu; pink sphere, P; yellow sphere, S; blue sphere, N; gray sphere, C. All hydrogen atoms are omitted for clarity.

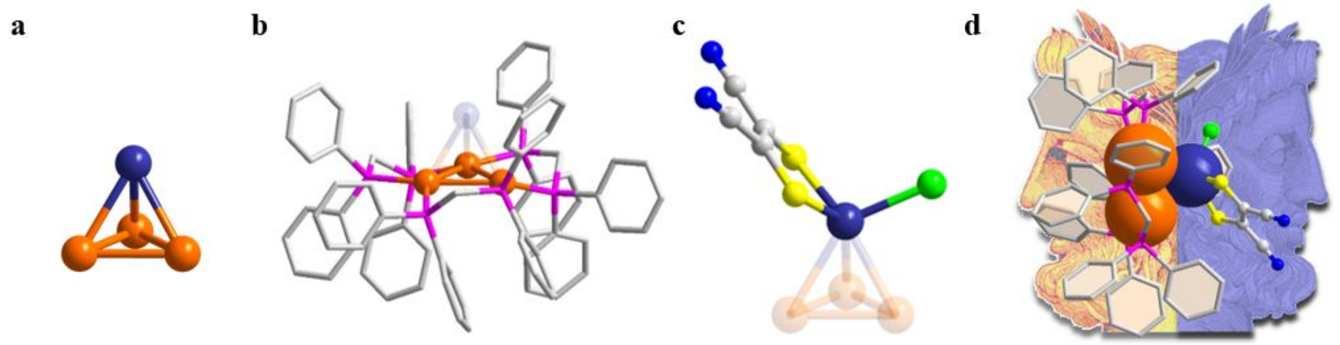

Figure S5. Structure of Janus $\mathbf{Au_3Cd}$. a. Au_3Cd. b. the Au-hemisphere in the Au_3Cd. c. the Cd-hemisphere in the Au_3Cd. d. molecular structure of Au_3Cd cluster. Indigo and yellow Janus image embodied Cd-hemisphere and Au hemisphere. Color legend: orange sphere, Au; Indigo sphere, Cd; pink sphere, P; yellow sphere, S; blue sphere, N; gray sphere, C. All hydrogen atoms are omitted for clarity.

Section S4 Characterizations.

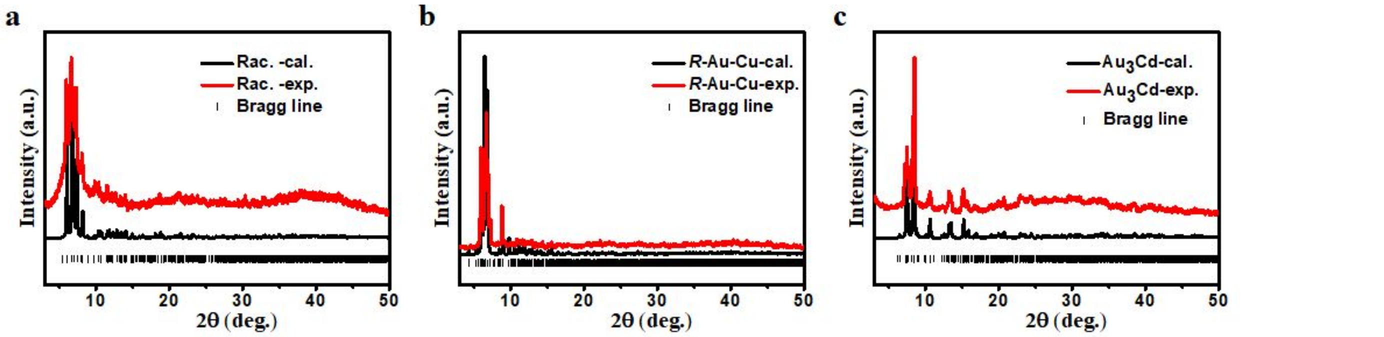

Figure S6. PXRD spectra of **racemate Au-Cu**, ***R*-Au-Cu** and **Au_3Cd**. a. PXRD spectra of **racemate Au-Cu**. b. PXRD spectra of ***R*-Au-Cu.** c. PXRD spectra of **Au_3Cd**.

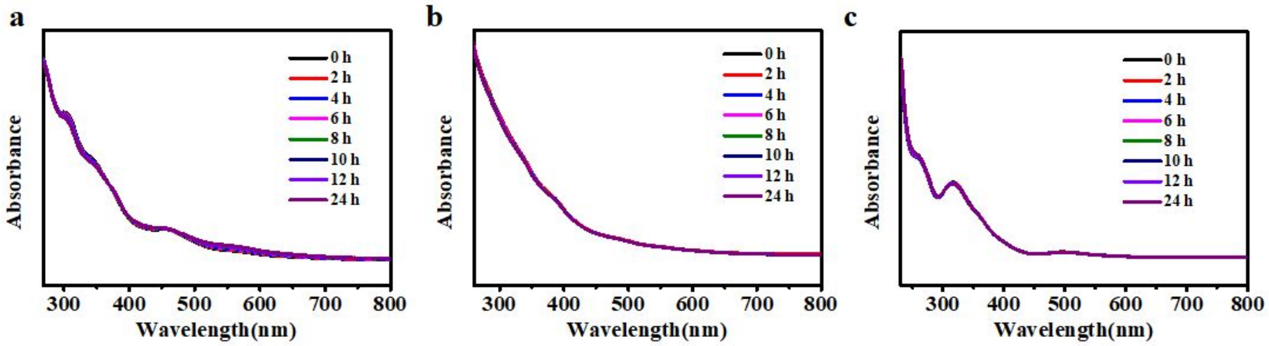

Figure S7. The time-dependent UV-vis absorption spectra of **racemate Au-Cu**, ***R*-Au-Cu** and **Au_3Cd**. a. The time-dependent UV-vis absorption spectra of **racemate Au-Cu** in DMF. b. The time-dependent UV-vis absorption spectra of ***R*-Au-Cu** in DCM**.** c. The time-dependent UV-vis absorption spectra of **Au_3Cd** in DCM.

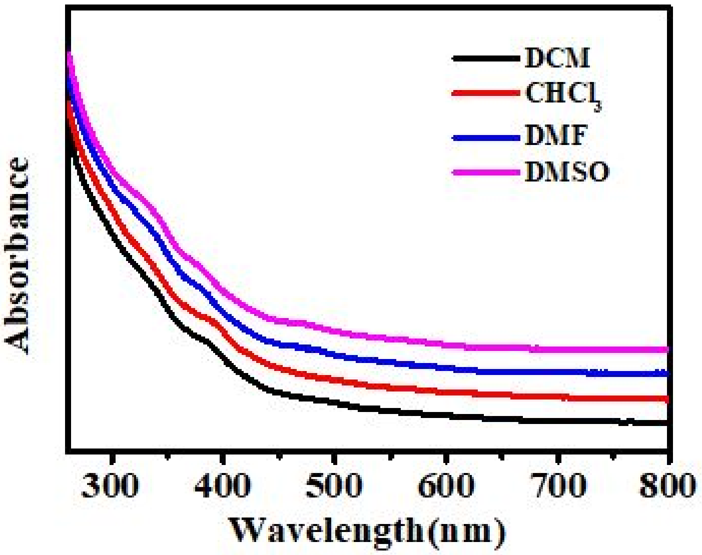

Figure S8. The UV-vis absorption spectra of ***R*-Au-Cu** in different solvents.

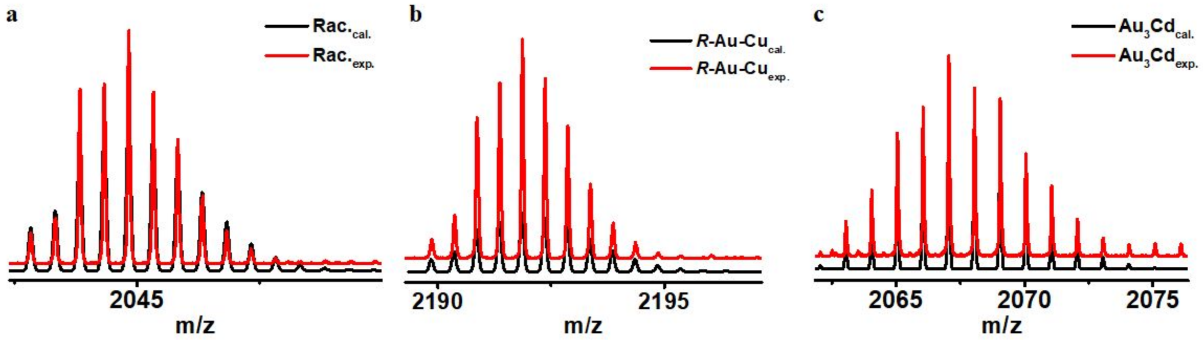

Figure S9. Mass spectra of **racemate Au-Cu**, ***R*-Au-Cu** in positive mode with Cs_2CO_3 and mass spectra of $\mathbf{Au_3Cd}$ in negative mode. a. The peak at m/z = 2044.83 corresponds to $[Cs_2Cu_4Au_8(TPP)_6(MNT)_3]^{2+}$. b. The peak at m/z = 2191.86 corresponds to $[Cs_2Cu_4Au_8(R\text{-}BINAP)_3(MNT)_3]^{2+}$. c. The peak at m/z = 2067.05 corresponds to $[Au_3Cd(DPPM)_3(MNT)Cl_2]^{-}$.

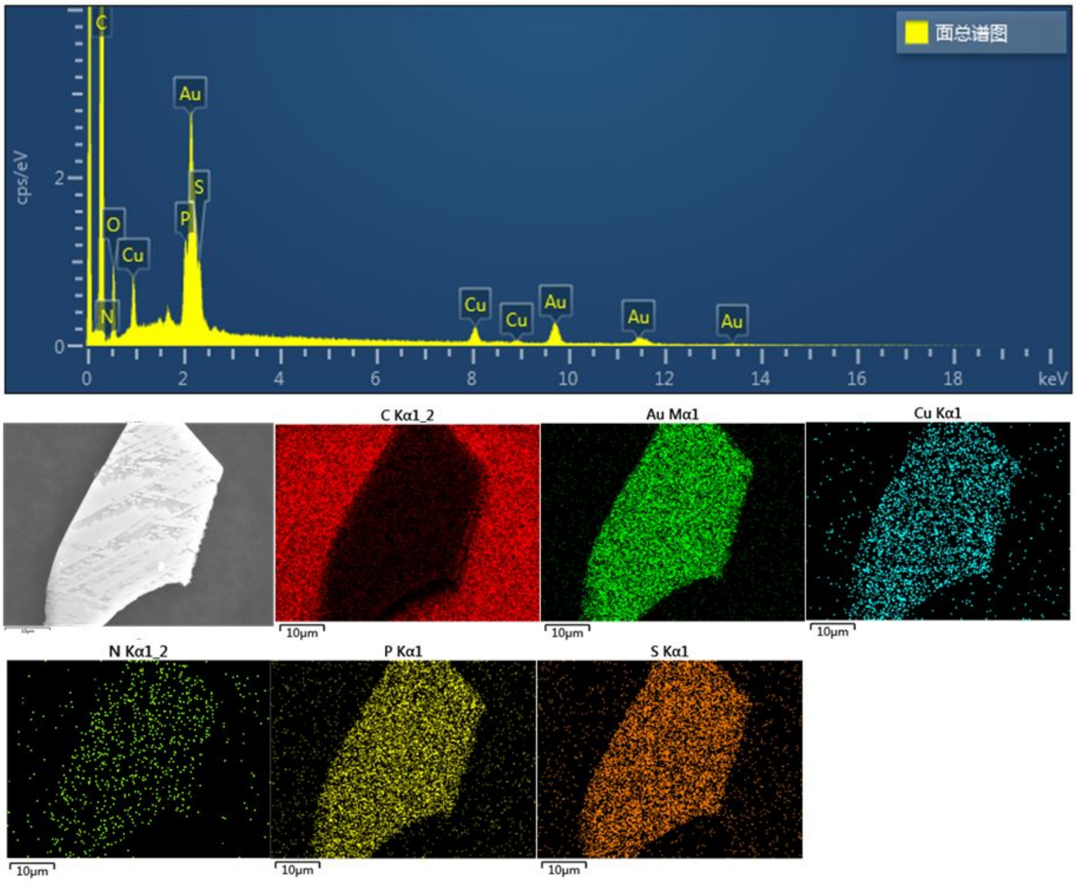

Figure S10. The EDS mapping of the **racemate Au-Cu** crystal. The result shows that there is no sodium or chlorine in **racemate Au-Cu**, indicating that the cluster is neutral.

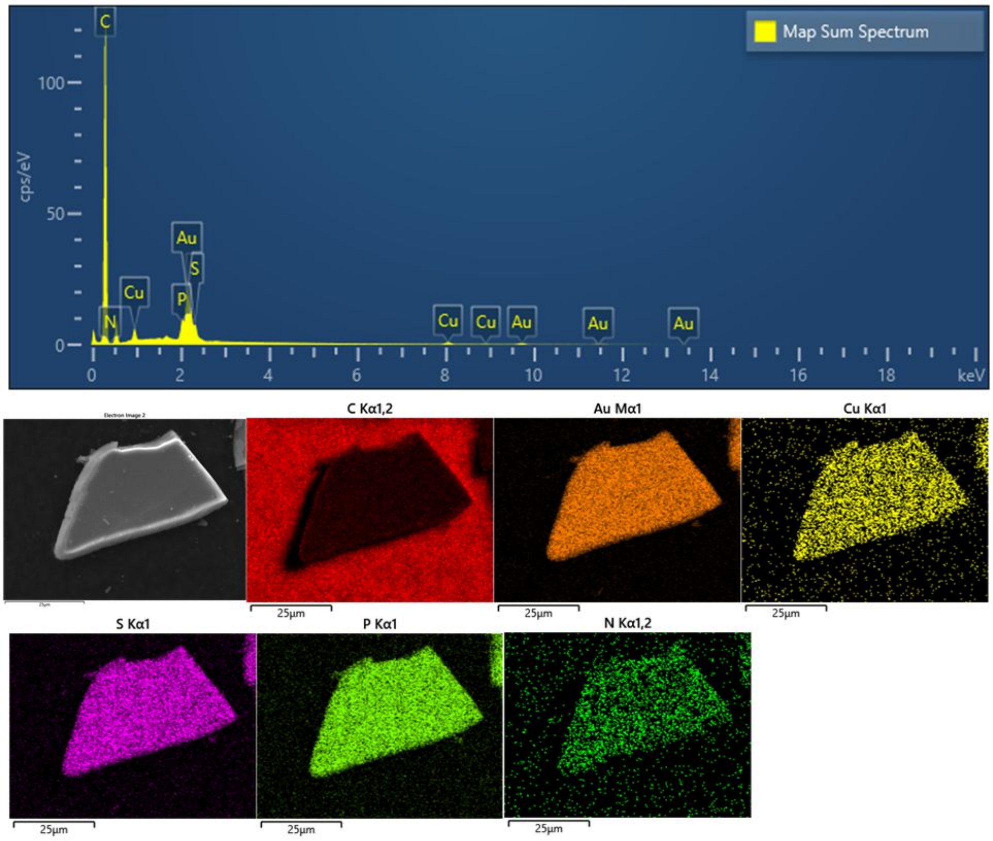

Figure S11. The EDS mapping of the ***R*-Au-Cu** crystal. The EDS elemental analysis result shows that there is no sodium or chlorine in ***R*-Au-Cu**, indicating that the cluster is neutral.

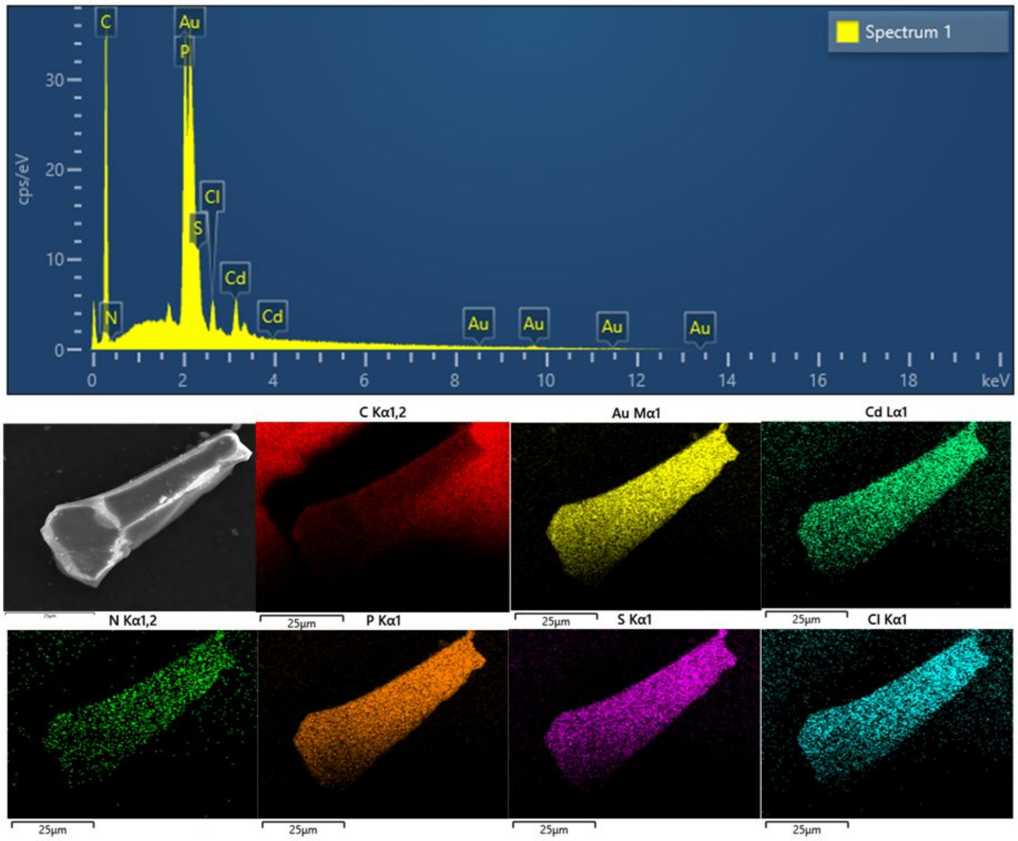

Figure S12. The EDS mapping of the **Au_3Cd** crystal.

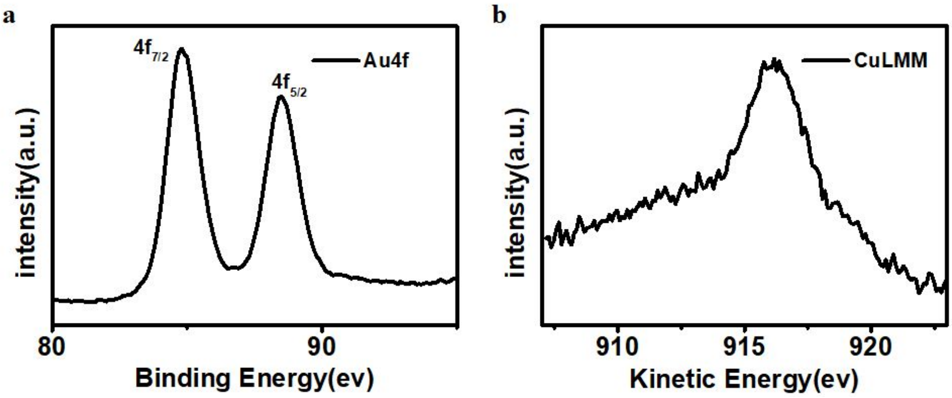

Figure S13. The XPS spectra of **racemate Au-Cu** nanocluster. a. Au4f. b. CuLMM. The XPS survey spectrum of Racemate Au-Cu showed an Au $4f_{7/2}$ (BE of 84.8 eV) peak, suggesting the presence of Au (0) in the cluster. The Cu LMM Auger spectrum of the Racemate Au-Cu shows one main peak at 916.17 eV, which are similar to those obtained for Cu−SR bonds in the Cu(I) oxidation state[5].

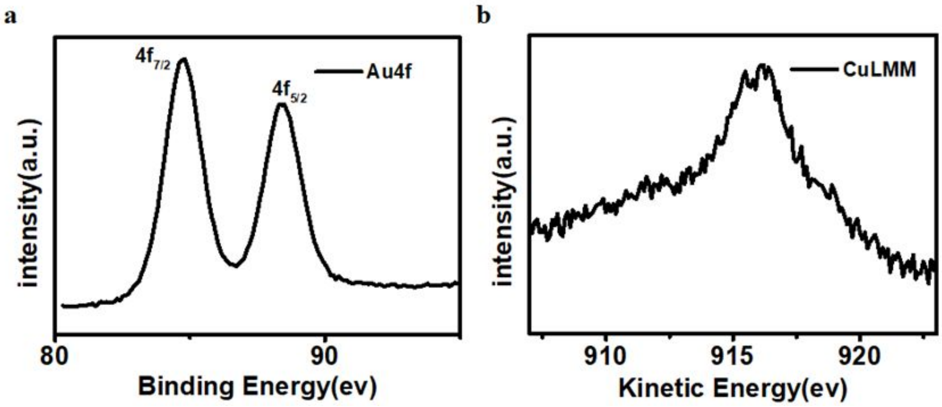

Figure S14. The XPS spectra of ***R*-Au-Cu** nanocluster. a. Au4f. b. CuLMM. The XPS survey spectrum of ***R*-Au-Cu** showed an Au $4f_{7/2}$ (BE of 84.8 eV) peak, suggesting the presence of Au (0) in the cluster. The Cu LMM Auger spectrum of the ***R*-Au-Cu** shows one main peak at 916.19 eV, which are similar to those obtained for Cu−SR bonds in the Cu(I) oxidation state[5].

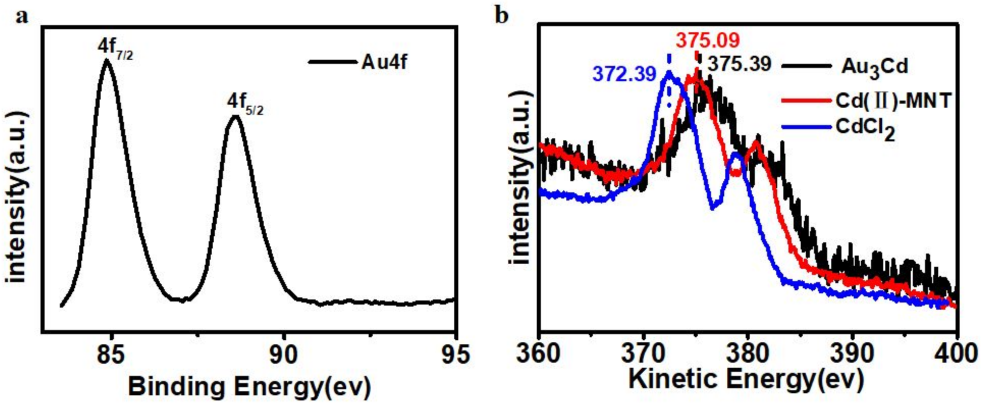

Figure S15. X-ray photoelectron spectrum (XPS) data of **Au_3Cd**: a) Au4f. b) CdMNN. The XPS spectra of **Au_3Cd** nanocluster. a. Au4f. b. CdMNN. The XPS survey spectrum of Au_3Cd showed an Au $4f_{7/2}$ (BE of 84.85 eV) peak, suggesting the presence of Au (0) in the cluster. The CdMNN Auger spectrum of the **Au_3Cd** shows one main peak at 375.39 eV accompanied by one shoulder at around 380.99 eV, which are similar to those obtained for Cd−MNT bonds in the Cd(II) oxidation state.

Section S5 Theoretical calculations

Density functional theory (DFT) and time-dependent density functional theory (TD-DFT) calculation were performed with Gaussian 16[6] under Perdew−Burke−Ernzerhof (PBE) functional[7]. All calculations were conducted using 6-31g** basis set for H, C, N , P ,S and Cl atoms[8], and SDD effective core potentials for Cu, Cd and Au atoms[9]. The single crystal structure was chosen as initial guess for ground state optimization and all reported stationary points were verified as true minima by the absence of negative eigenvalues in the vibrational frequency analysis. The calculated absorption spectra and CD spectra were obtained from by Multiwfn 3.7 (dev)[10].

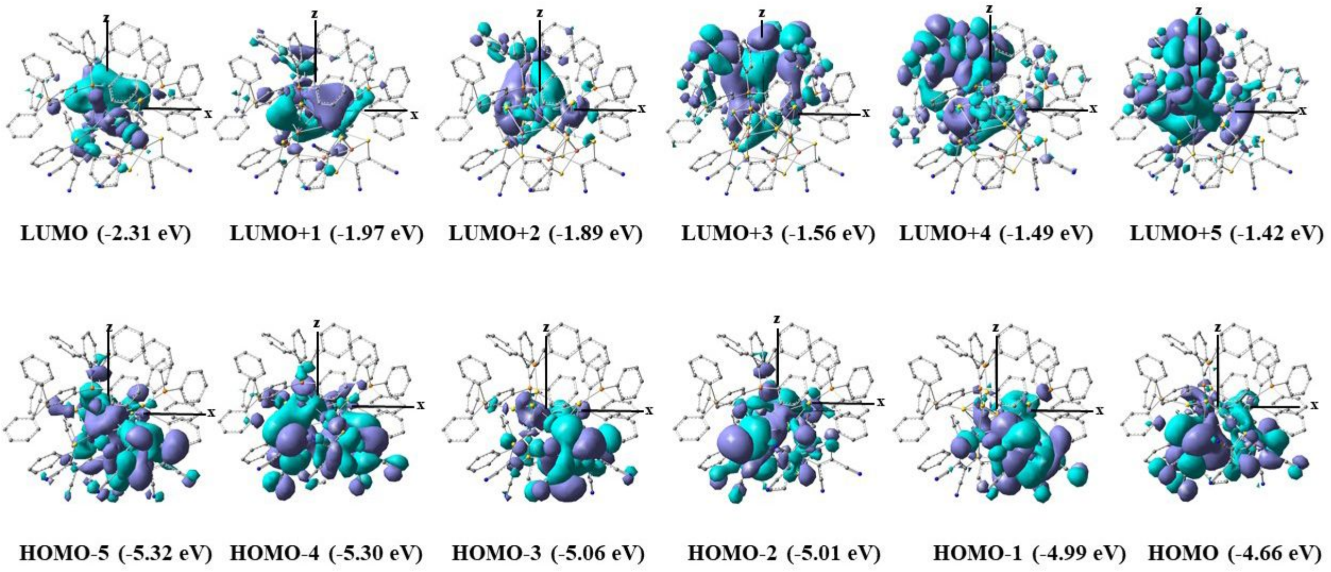

Figure S16. Selected frontier MO representations for *R*-cluster component in **racemate Au-Cu**.

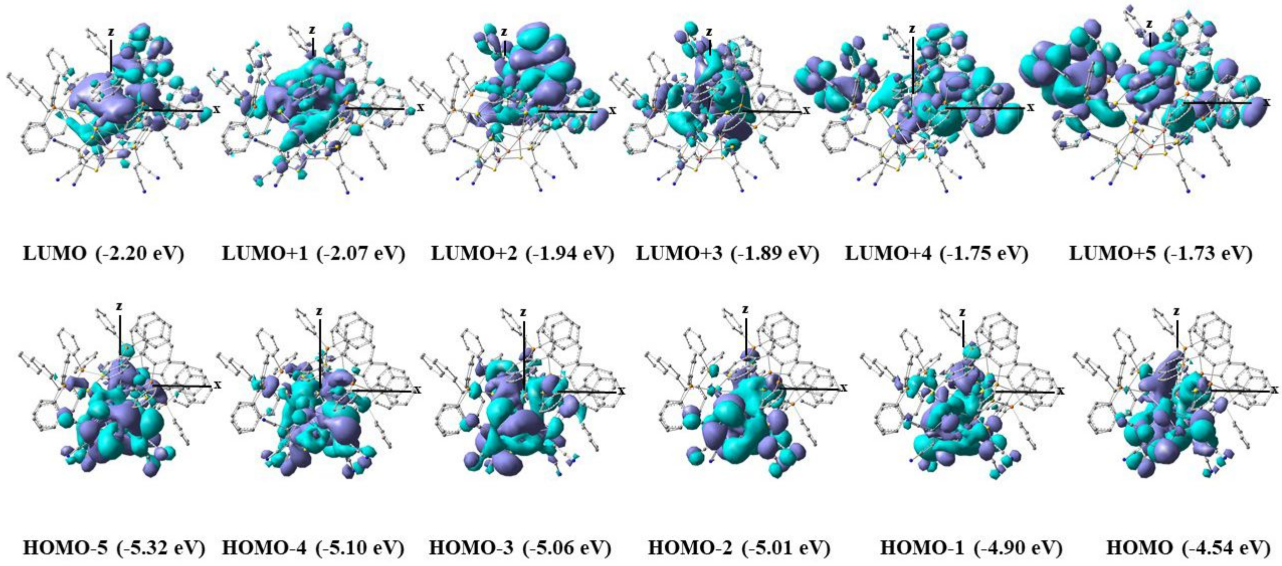

Figure S17. Selected frontier MO representations for ***R*-Au-Cu** cluster.

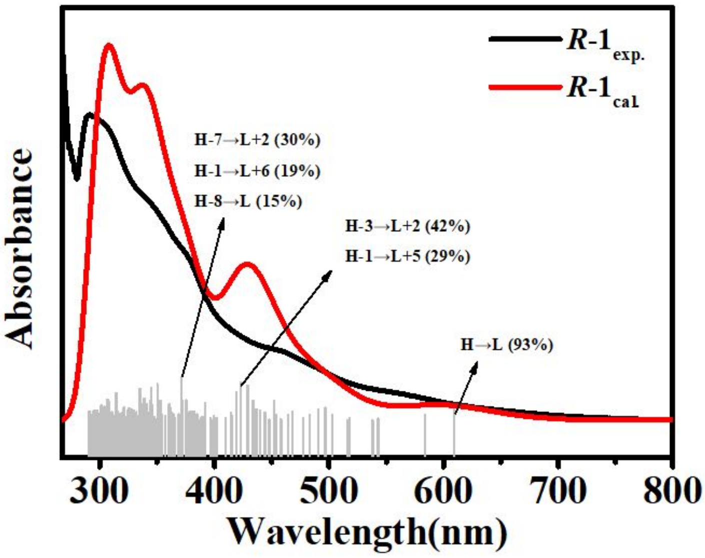

Figure S18. Experimental and Calculated UV−Vis spectra of **racemate Au-Cu** (***R*-1**). Owing to strong quantum-size effects, the UV−vis absorption spectra of ***R*-1** shows multiple molecular-like single-electron transitions, which is consistent with the calculated result. The assignments of transitions can be found in Table S4.

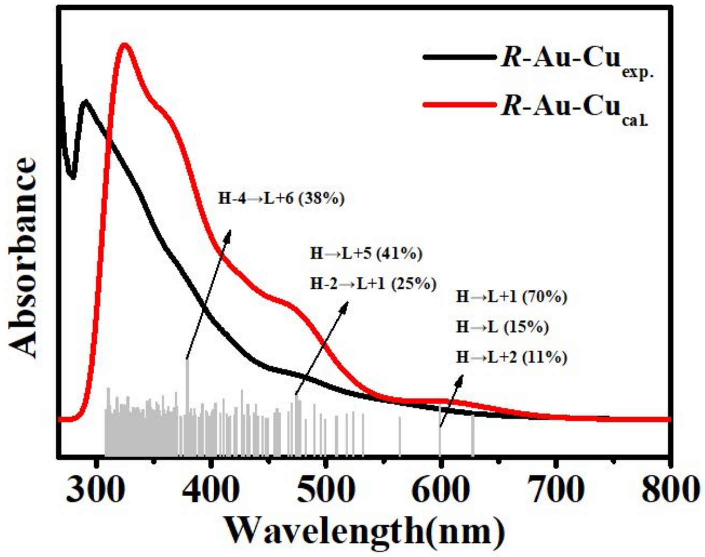

Figure S19. Experimental and Calculated UV−Vis spectra of ***R*-Au-Cu**. Owing to strong quantum-size effects, the UV−vis absorption spectra of ***R*-Au-Cu** shows multiple molecular-like single-electron transitions, which is consistent with the calculated result. The assignments of transitions can be found in Table S5.

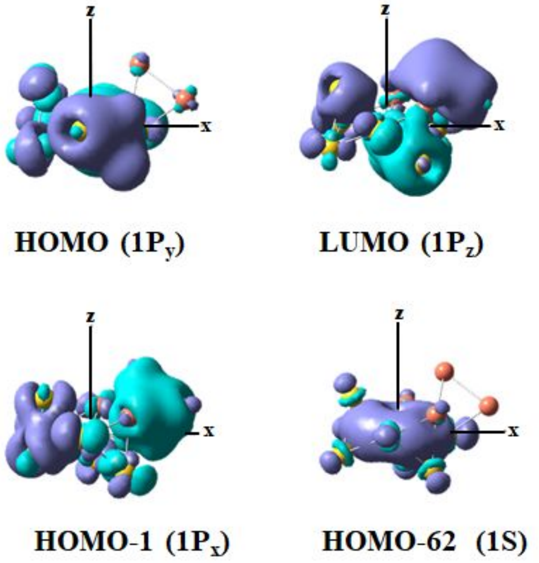

Figure S20. The Superatomic orbitals of metal skeleton $[Au_8Cu_4]^{6+}$ (6e) of *R*-cluster component in **racemate Au-Cu** cluster.

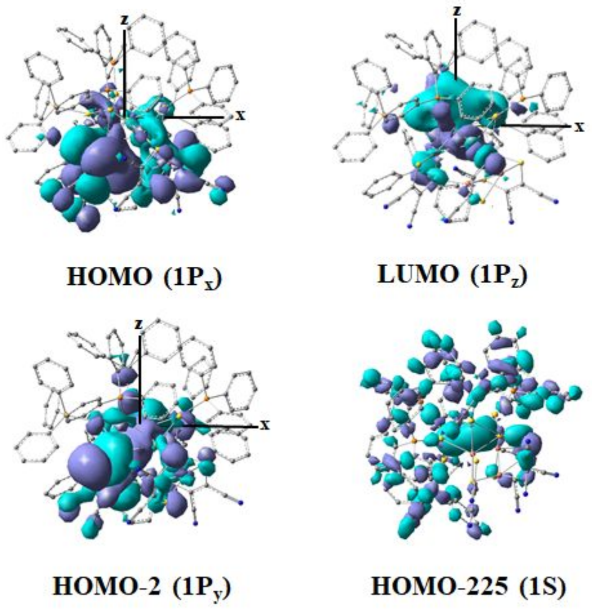

Figure S21. Superatomic orbitals in the *R*-cluster component of **Racemate Au-Cu**.

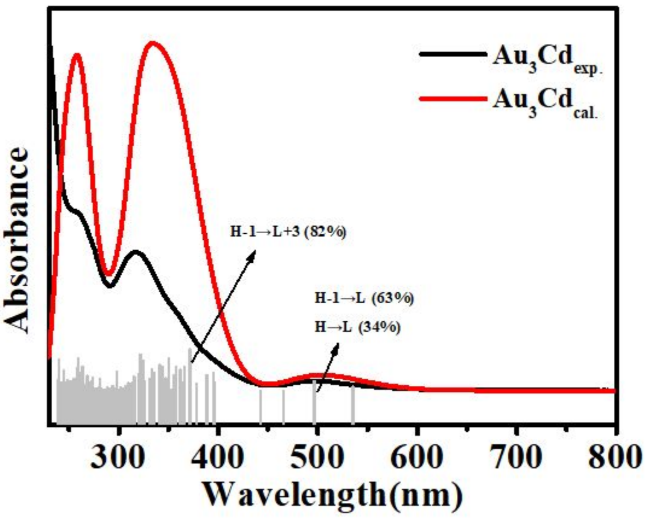

Figure S22. Experimental and Calculated UV−Vis spectra of **Au_3Cd**. Owing to strong quantum-size effects, the UV−vis absorption spectra of **Au_3Cd** shows multiple molecular-like single-electron transitions, which is consistent with the calculated result. the assignments of transitions can be found in Table S6.

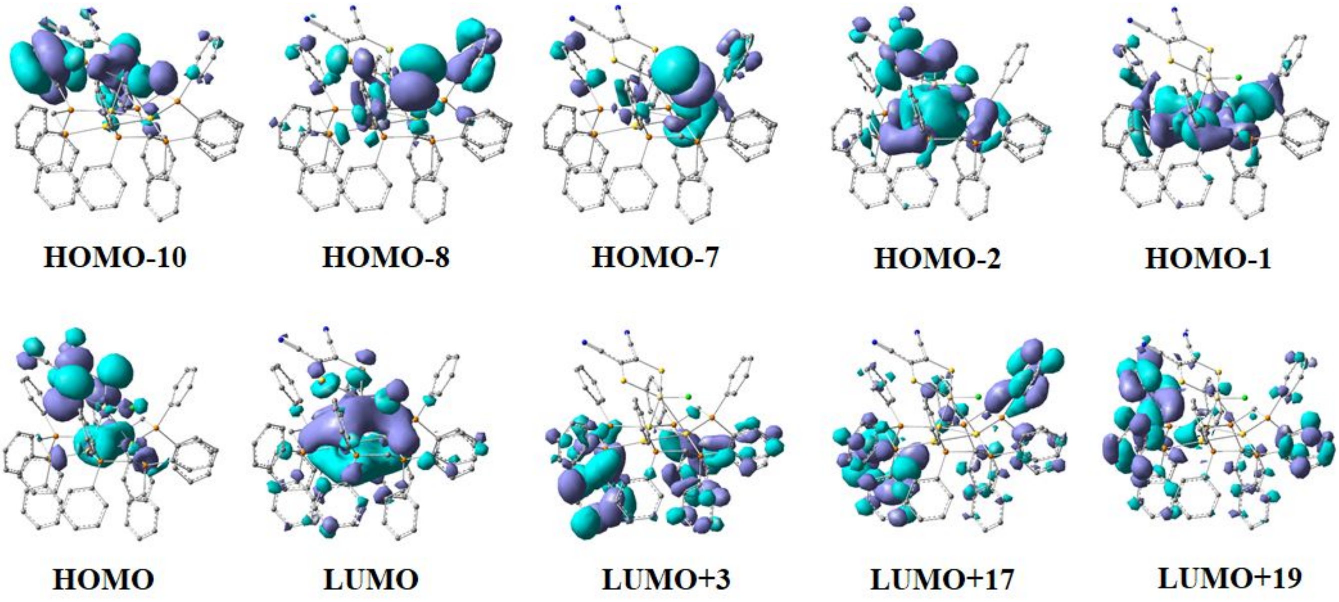

Figure S23. Selected frontier MO of the complete **Au_3Cd** cluster.

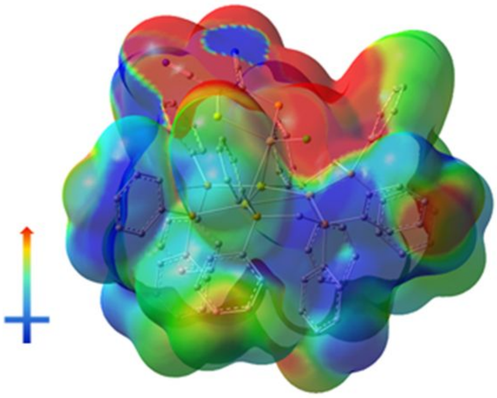

Figure S24. Dipole moment. Direction and value of dipole moment in **Au_3Cd**. The red represents the electron-rich region and the blue denoting electron-poor region.

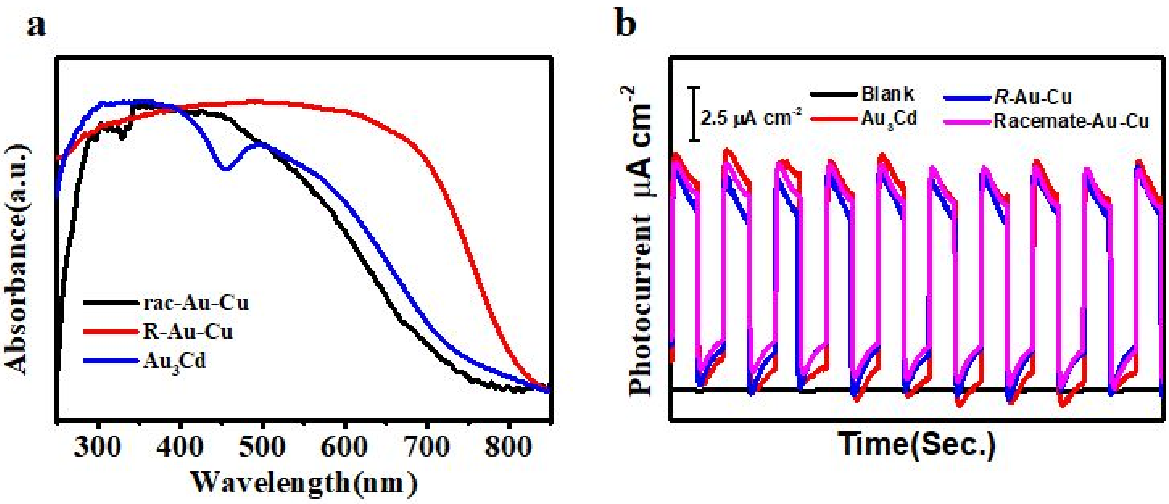

Figure S25. UV-visible diffuse reflectance spectra and Photocurrent Response Properties of these Janus clusters. a. Normalized UV-visible diffuse reflectance spectra of **racemate-Au-Cu**, ***R*-Au-Cu** and **Au_3Cd** in the solid state. b. Visible light driven photocurrent responses of blank (black), **Au_3Cd** (red), ***R*-Au-Cu** (blue) and **racemate-Au-Cu** (pink) in a 0.1 M KCl aqueous solution under repetitive Xe lamp irradiation (intervals of 10 s; $\lambda > 420$ nm).

Considering that MNT^{2-} ligands have delocalized π bonds, the electron density can be enhanced, which is beneficial to increase the electron transport rate. In addition, these clusters have large dipole moments, small band gaps and excellent optical absorption properties,[11,12] which make them potential hybrid organic-inorganic optoelectronic materials. Therefore, we examined the optoelectronics properties of **racemate-Au-Cu**, ***R*-Au-Cu** and **Au_3Cd** (Fig. S26). We performed the photocurrent response tests in a 0.1 M KCl aqueous solution through a typical three-electrode system keeping the bias voltage at 0.3 V (Ag/AgCl as the reference electrode, Pt wire as the assisting electrode, glassy carbon electrode as the working electrode). Upon switching on/off Xe lamp (intervals of 10 s; $\lambda > 420$ nm; 300 W), the photocurrent density reversibly increases and decreases correspondingly, indicating a good generation and separation efficiency of photoinduced electron/hole pairs. The photocurrent response has good reproducibility.

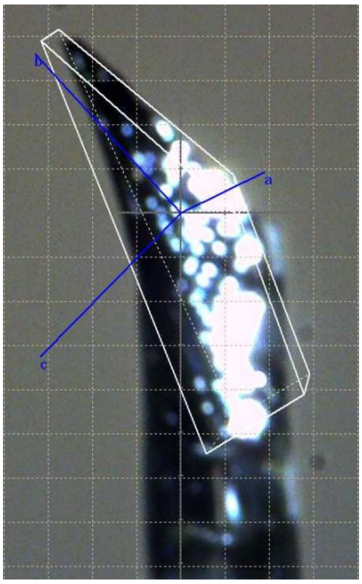

Figure S26. Determination of the coordinate axis of the ***R*-Au-Cu** crystal.

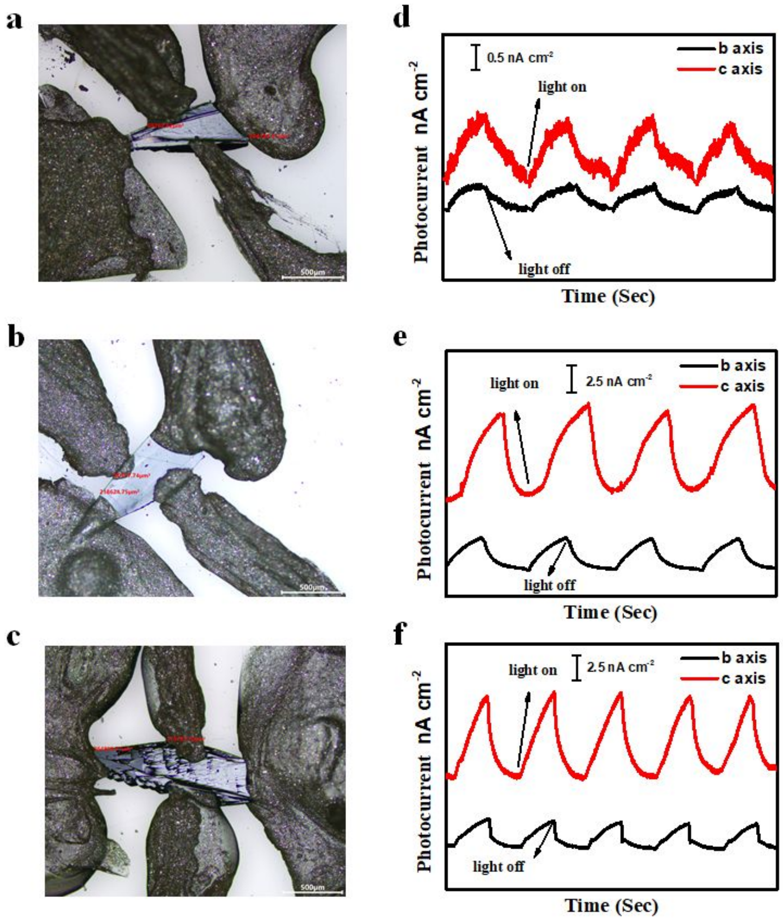

Figure S27. (a-c) Configurations of single-crystals in the measurement system for ***R*-Au-Cu** and (d-f) ***R*-Au-Cu** single-crystal device under dark and 450 nm light illumination with a power density of 1 mW/cm2 at 5 V bias.

We test the anisotropic photoelectric response by using three single crystals of ***R*-Au-Cu**, which is enough large for operation. When switching on/off the laser (bias voltage at 5 V; intervals of 10 s; λ = 450 nm), the photocurrent density reversibly increases and decreases correspondingly, indicating a good generation and separation efficiency of photoinduced electron/hole pairs. We observe that the photocurrent densities along the *c*-axis for these three single-crystal devices are 2.7, 2.9, and 3.1 times higher than those along the *b*-axis, respectively. Considering that the inter-cluster spacing along the *c*-axis is 1.61 nm, which is smaller than the 2.68 nm along the *b*-axis, and the dipole interactions are inversely proportional to the cube of cluster spacing,[13] we tentatively ascribed the anisotropic photo-electronic response to the distinct dipole interactions along the different direction.

Table S1. Crystal data and structure refinement of **Racemate Au-Cu**, ***R*-Au-Cu**, ***S*-Au-Cu**, and **Au_3Cd** nanoclusters.

Identification code	**Racemate Au-Cu**	***R*-Au-Cu**	***S*-Au-Cu**	**Au_3Cd**
Empirical formula	$C_{120.5}H_{91}Au_8ClCu_4N_6P_6S_6$	$C_{291}H_{198}Au_{16}Cl_6Cu_8N_{12}P_{12}S_{12}$	$C_{144}H_{96}Au_8Cu_4N_6P_6S_6$	$C_{80}H_{70}Au_3CdClN_2OP_6S_2$
CCDC	2151533	2151535	2151534	2151532
Formula weight	3866.51	8491.45	4118.33	2064.07
Temperature/K	200.00(10)	100.00(10)	200.00(10)	200.00(10)
Crystal system	monoclinic	monoclinic	monoclinic	orthorhombic
Space group	*Pn*	$P2_1$	$P2_1$	*Pbca*
a/Å	17.27050(10)	16.57420(10)	16.6336(2)	22.9702(2)
b/Å	16.42320(10)	26.81340(10)	26.8950(3)	23.8042(2)
c/Å	22.28820(10)	31.74440(10)	31.7629(4)	27.0023(3)
α/°	90	90	90	90
β/°	99.25	91.4170(10)	91.3460(10)	90
γ/°	90	90	90	90
V/Å^3	6239.57(6)	14103.24(11)	14205.6(3)	14764.5(2)
Z	2	2	4	8
ρ_{calc}g/cm^3	2.058	2.000	1.926	1.857
μ/mm^{-1}	20.141	18.239	17.577	15.689
F(000)	3614.0	7996.0	7744.0	7968.0
Crystal size/mm^3	0.15 × 0.15 × 0.1	0.2 × 0.2 × 0.12	0.2 × 0.12 × 0.12	0.2 × 0.2 × 0.15
Radiation	CuKα (λ = 1.54184)	CuKα (λ = 1.54184)	CuKα (λ = 1.54184)	CuKα (λ = 1.54184)
2θ range for data collection /°	5.382 to 148.218	5.57 to 147.55	5.314 to 148.254	6.27 to 148.768
Index ranges	$-21 \le h \le 18$, $-20 \le k \le 19$, $-27 \le l \le 27$	$-20 \le h \le 18$, $-31 \le k \le 33$, $-38 \le l \le 39$	$-20 \le h \le 11$, $-33 \le k \le 32$, $-39 \le l \le 37$	$-28 \le h \le 26$, $-27 \le k \le 29$, $-32 \le l \le 31$
Reflections collected	76273	160012	123404	53723
Independent reflections	19719 [R_{int} = 0.0394, R_{sigma} = 0.0356]	54572 [R_{int} = 0.0481, R_{sigma} = 0.0494]	53322 [R_{int} = 0.0688, R_{sigma} = 0.0908]	14686 [R_{int} =0.0481, R_{sigma} = 0.0426]
Data/restraints/parameters	19719/476/1552	54572/313/3190	53322/651/3121	14686/214/959
Goodness-of-fit on F^2	1.073	1.051	1.043	1.029
Final R indexes [I>=2σ (I)]	R_1 = 0.0351, wR_2 = 0.0900	R_1 = 0.0519, wR_2 = 0.1399	R_1 = 0.0808, wR_2 = 0.2088	R1 = 0.0554, wR2 = 0.1433
Final R indexes [all data]	R_1 = 0.0365, wR_2 = 0.0911	R_1 = 0.0541, wR_2 = 0.1416	R_1 = 0.0994, wR_2 = 0.2239	R1 = 0.0688, wR2 = 0.1551
Largest diff. peak/hole / e Å^{-3}	1.51/-2.49	3.54/-2.89	5.75/-2.52	3.58/-1.61
Flack parameter	-0.019(4)	-0.010(3)	-0.019(7)	

Table S2. The Au-Cu bond length (Å) in the interface of ***R*-1** and ***R*-Au-Cu**.

***R*-1**		***R*-Au-Cu**	
Au6-Cu1	2.7279(19)	Au6-Cu1	2.693(3)
Au2-Cu1	2.742(2)	Au2-Cu1	2.614(3)
Au1-Cu1	2.6191(19)	Au1-Cu1	2.687(3)
Au7-Cu1	2.6196(19)	Au7-Cu1	2.662(3)
Au5-Cu2	2.577(2)	Au5-Cu2	2.630(3)
Au7-Cu2	2.706(2)	Au7-Cu2	2.522(3)
Au6-Cu2	2.6955(19)	Au6-Cu2	2.647(3)
Au4-Cu2	2.769(2)	None	None

Table S3 Comparison of bond angles (˚) of the Cu_4Au_8 core in ***R*-1 and *R*-Au-Cu.**

Au6Au1Au2	106.20(2)	None	None
Au7Au5Au6	57.323(19)	Au6Au5Au7	56.95(2)
Au7Au1Au2	56.970(17)	Au2Au1Au7	60.39(2)
Au7Au1Au6	56.828(18)	None	None
Au7Au5Au4	56.161(18)	Au4Au5Au7	57.07(2)
Cu1Au1Au2	60.08(4)	Cu1Au1Au2	57.62(6)
Cu1Au1Au6	59.78(4)	None	None
Cu1Au1Au7	57.98(4)	Cu1Au1Au7	58.70(6)
Au1Au2Au3	117.35(2)	Au1Au2Au3	100.77(3)
Au1Au2Au8	93.64(2)	None	None
Au3Au2Au8	54.237(17)	None	None
Au7Au2Au1	60.281(19)	Au1Au2Au7	59.94(2)
Au7Au2Au3	57.128(17)	Au7Au2Au3	59.57(2)
Au7Au2Au8	57.115(19)	None	None
Au7Au2Cu1	57.75(4)	Cu1Au2Au7	59.37(6)
Cu1Au2Au1	55.89(4)	Cu1Au2Au1	60.25(6)
Cu1Au2Au3	87.93(4)	Cu1Au2Au3	116.77(6)
Cu1Au2Au8	114.79(4)	None	None
Au2Au3Au4	106.68(2)	Au2Au3Au4	118.35(3)
Au4Au7Au6	116.32(3)	Au6Au7Au4	120.29(3)

Au7Au3Au2	56.722(17)	Au7Au3Au2	59.73(2)
Au6Au7Au2	116.15(2)	Au6Au7Au2	117.08(3)
Au7Au3Au4	54.954(17)	Au7Au3Au4	58.73(2)
Au8Au3Au2	63.619(19)	Au8Au3Au2	89.28(3)
Au8Au3Au4	62.52(2)	Au8Au3Au4	62.13(3)
Au4Au7Au2	127.46(2)	Au4Au7Au2	122.03(3)
Au8Au3Au7	60.53(2)	Au8Au3Au7	58.72(2)
Au4Au7Au1	151.91(3)	Au4Au7Au1	137.85(3)
Au5Au4Au3	115.00(2)	Au3Au4Au5	121.71(3)
Au2Au7Au5	149.05(3)	Au2Au7Au5	173.80(3)
Au5Au4Au8	91.37(2)	Au8Au4Au5	92.84(3)
Au7Au4Au3	55.868(17)	Au7Au4Au3	59.81(2)
Au7Au4Au5	59.132(19)	Au7Au4Au5	61.94(2)
Au6Au7Au8	153.91(3)	Au8Au7Au6	125.10(3)
Au7Au4Au8	57.031(19)	Au7Au4Au8	57.40(2)
Au4Au7Au8	67.54(2)	Au8Au7Au4	63.43(3)
Au7Au4Cu2	59.71(4)	None	None
Au2Au7Au8	66.84(2)	Au8Au7Au2	90.33(3)
Au8Au4Au3	53.005(17)	Au3Au4Au8	57.14(2)
Au8Au7Au1	101.12(2)	Au8Au7Au1	74.65(3)
Au8Au7Au5	100.87(3)	Au8Au7Au5	95.85(3)
Cu2Au4Au3	92.19(4)	None	None
Au5Au7Au1	94.13(2)	Au1Au7Au5	122.48(3)
Cu2Au4Au5	54.00(4)	None	None
Cu2Au4Au8	116.72(4)	None	None
Au6Au5Au4	104.88(2)	Au6Au5Au4	113.96(3)
Au6Au7Cu2	60.12(4)	Cu2Au7Au6	61.16(6)
Cu2Au5Au4	60.39(4)	Cu2Au5Au4	81.41(6)
Au4Au7Cu2	62.06(4)	Cu2Au7Au4	85.68(7)
Cu2Au5Au6	59.72(4)	Cu2Au5Au6	58.94(6)
Au2Au7Cu2	153.13(5)	Cu2Au7Au2	115.95(7)
Cu2Au5Au7	60.91(4)	Cu2Au5Au7	54.49(6)
Au1Au6Cu3	115.28(4)	None	None
Au2Au7Au1	62.748(19)	Au1Au7Au2	59.67(2)

Au5Au6Au1	91.34(3)	None	None
Cu1Au7Cu2	97.06(6)	Cu2Au7Cu1	79.44(9)
Au5Au6Cu3	112.23(5)	Au5Au6Cu3	109.20(6)
Cu1Au7Au5	124.56(5)	Cu1Au7Au5	117.58(6)
Au7Au6Au1	60.398(19)	None	None
Cu1Au7Au3	95.78(4)	Cu1Au7Au3	116.20(6)
Au7Au6Au5	60.08(2)	Au7Au6Au5	63.70(2)
Cu1Au7Au6	62.02(4)	Cu1Au7Au6	60.62(6)
Au7Au6Cu1	58.00(4)	Au7Au6Cu1	59.46(6)
Au7Au6Cu2	60.53(4)	Cu2Au6Au7	56.58(6)
Au7Au6Cu3	80.47(4)	Au7Au6Cu3	94.12(6)
Au7Au6Cu4	119.38(5)	Au7Au6Cu4	120.01(6)
Cu1Au7Au4	149.13(5)	Cu1Au7Au4	161.87(7)
Cu1Au7Au2	62.27(5)	Cu1Au7Au2	57.66(6)
Cu1Au7Au8	129.01(5)	Cu1Au7Au8	132.71(7)
Cu1Au7Au1	57.96(4)	Cu1Au7Au1	59.60(6)
Cu4Au6Cu1	62.40(6)	Cu1Au6Cu4	60.69(8)
Cu1Au6Au1	56.06(4)	None	None
Cu1Au6Au5	118.03(4)	Cu1Au6Au5	120.60(6)
Cu1Au6Cu3	59.70(6)	Cu1Au6Cu3	60.96(8)
Cu2Au6Au1	120.80(5)	None	None
Cu2Au6Au5	55.64(5)	Cu2Au6Au5	58.36(6)
Cu2Au6Cu1	94.76(6)	Cu2Au6Cu1	76.72(8)
Cu2Au6Cu3	57.25(6)	Cu2Au6Cu3	54.22(8)
Cu2Au6Cu4	116.70(7)	Cu2Au6Cu4	112.32(9)
Cu4Au6Au1	94.96(5)	None	None
Cu4Au6Au5	172.08(5)	Cu4Au6Au5	167.35(7)
Cu4Au6Cu3	60.61(6)	Cu4Au6Cu3	59.47(8)
Au2Au7Au3	66.149(19)	None	None
Au3Au7Au1	128.82(3)	Au1Au7Au3	101.37(3)
Au3Au7Au5	133.88(2)	Au3Au7Au5	122.42(3)
Au3Au7Au8	59.705(19)	Au8Au7Au3	59.55(2)
Au3Au7Cu2	101.81(4)	Cu2Au7Au3	115.51(6)
Au4Au7Au3	69.178(19)	Au4Au7Au3	61.46(2)

Au4Au7Au5	64.71(2)	Au4Au7Au5	61.00(2)
None	None	Au3Au8Au1	90.14(3)
Au6Au7Au1	62.77(2)	Au6Au7Au1	80.13(3)
Au3Au8Au7	59.760(19)	Au7Au8Au3	61.73(2)
Au6Au7Au3	146.35(3)	Au6Au7Au3	175.30(4)
Au6Au7Au5	62.60(2)	Au6Au7Au5	59.36(2)
None	None	Au7Au8Au1	53.70(2)
None	None	Au4Au8Au1	112.73(3)
None	None	Au7Au1Au8	51.65(2)
None	None	Au2Au1Au8	78.91(2)
None	None	Cu1Au1Au8	109.40(6)
Au5Cu2Au7	62.78(5)	Au7Cu2Au5	67.41(6)
Au6Cu2Au7	59.35(4)	Au7Cu2Au6	62.27(6)
Au7Cu2Cu3	85.00(7)	Au7Cu2Cu3	109.26(12)
Au5Cu2Cu3	131.03(9)	Cu3Cu2Au5	128.14(12)
Cu2Au7Au1	122.76(4)	Cu2Au7Au1	134.15(7)
Au6Cu2Cu3	67.36(6)	Cu3Cu2Au6	70.49(9)
Cu2Au7Au5	56.31(5)	Cu2Au7Au5	58.10(6)
Cu2Au7Au8	129.59(5)	Cu2Au7Au8	147.55(7)
Au2Au8Au4	107.01(2)	None	None
Au3Au8Au2	62.144(19)	None	None
Au3Au8Au4	64.47(2)	Au3Au8Au4	60.72(2)
Au7Au8Au2	56.041(18)	None	None
Au7Au8Au4	55.424(19)	Au7Au8Au4	59.17(2)
Cu1Cu3Cu4	58.27(7)	Cu4Cu3Cu1	56.56(9)
Au1Cu1Au2	64.03(5)	Au2Cu1Au1	62.13(6)
Au1Cu1Au6	64.16(4)	Au1Cu1Au6	80.81(8)
Au1Cu1Au7	64.05(4)	Au7Cu1Au1	61.70(6)
Au1Cu1Cu3	128.76(8)	Au1Cu1Cu3	146.02(12)
Au1Cu1Cu4	97.48(7)	Au1Cu1Cu4	105.08(11)
Au6Cu1Cu4	58.28(6)	Au6Cu1Cu4	60.54(8)
Au2Cu1Cu3	132.78(8)	Au2Cu1Cu3	134.48(11)
Au6Cu1Cu3	65.25(6)	Au6Cu1Cu3	65.30(8)
Au2Cu1Cu4	161.06(9)	Au2Cu1Cu4	164.59(13)

Au6Cu1Au2	112.45(7)	Au2Cu1Au6	121.57(10)
Au7Cu1Au2	59.98(4)	Au2Cu1Au7	62.97(6)
Au7Cu1Au6	59.98(4)	Au7Cu1Au6	59.92(6)
Au7Cu1Cu3	84.25(7)	Au7Cu1Cu3	97.07(10)
Au7Cu1Cu4	117.28(8)	Au7Cu1Cu4	120.33(11)
Cu4Cu1Cu3	61.34(7)	Cu4Cu1Cu3	60.92(9)
Au5Cu2Au6	64.65(5)	Au5Cu2Au6	62.70(6)
Au6Cu2Au4	112.20(7)	None	None
Au7Cu2Au4	58.23(4)	None	None
Cu3Cu2Au4	127.99(8)	None	None
Cu1Cu3Au6	55.05(6)	Cu1Cu3Au6	53.74(7)
Cu2Cu3Au6	55.40(6)	Cu2Cu3Au6	55.29(8)
Cu2Cu3Cu1	90.31(9)	Cu2Cu3Cu1	73.37(10)
Cu2Cu3Cu4	108.56(9)	Cu2Cu3Cu4	109.20(12)
Cu4Cu3Au6	54.16(6)	Cu4Cu3Au6	55.21(8)
Au6Cu4Cu1	59.31(6)	Au6Cu4Cu1	58.76(8)
Au6Cu4Cu3	65.23(6)	Au6Cu4Cu3	65.32(8)
Cu1Cu4Cu3	60.39(7)	Cu1Cu4Cu3	62.52(10)

Table S4 The absorptions of **racemate Au-Cu (*R*)** cluster calculated according to TDDFT method (H = HOMO, L = LUMO).

Wavelength (nm)	Osc. Strength	Major contribs
608.925	0.03	H->L (93%)
583.478	0.0172	H-1->L (88%)
452.213	0.0534	H-1->L+2 (21%), H-1->L+3 (23%)
429.186	0.0943	H-4->L+2 (25%), H-2->L+3 (14%), H-1->L+3 (21%)
422.862	0.1019	H-3->L+2 (42%), H-1->L+5 (29%)
371.129	0.1177	H-8->L (15%), H-7->L+2 (30%), H-1->L+6 (19%)

Table S5 Absorptions of ***R*-Au-Cu** cluster calculated according to TDDFT method (H = HOMO, L = LUMO).

Wavelength (nm)	Osc. Strength	Major contribs
598.895	0.04	H->L (15%), H->L+1 (70%), H->L+2 (11%)
489.665	0.0418	H-2->L (10%), H->L+4 (44%), H->L+6 (11%)

476.969	0.0513	H-2->L+1 (34%), H->L+5 (20%)
474.087	0.0762	H-2->L+1 (25%), H->L+5 (41%)
426.616	0.0765	H-3->L+3 (11%), H-2->L+3 (10%), H-1->L+4 (21%), H-1->L+6 (19%)
379.142	0.1734	H-4->L+6 (38%)

Table S6. Absorptions of **Au_3Cd** cluster calculated according to TDDFT method (H = HOMO, L = LUMO).

Wavelength (nm)	Osc. Strength	Major contribs
535.195	0.0128	H-1->L (34%), H->L (63%)
496.568	0.0283	H-1->L (63%), H->L (34%)
371.608	0.1203	H-1->L+3 (82%)
321.650	0.105	H->L+17 (82%)
259.254	0.0943	H-10->L (13%), H-8->L (20%), H-2->L+19 (14%)

Reference:

(1) CrysAlisPro 2012, Agilent Technologies. Version 1.171.36.31.

(2) Sheldrick, G. M. *Acta Cryst. A* **2015**, *71*, 3-8.

(3) Dolomanov, O. V.; Bourhis, L. J.; Gildea, R. J.; Howard, J. A. K.; Puschmann, H. *J. Appl. Cryst.* **2009**, *42*, 339-341.

(4) Spek, A. L. *Acta Cryst. C* **2015**, *71*, 9-18.

(5) Caprioli, F.; Decker, F.; Marrani, A. G.; Beccari, M.; Castro, V. D. *Phys. Chem. Chem. Phys*. **2010**, *12*, 9230−9238.

(6) Frisch, M. J. et al. *Gaussian, Inc., Wallingford CT*, **2016**.

(7) Perdew, J. P.; Burke, K.; Ernzerhof, M. *Phys. Rev. Lett*. **1996**, *77*, 3865.

(8) a) Petersson, G. A.; Bennett, A.; Tensfeldt, T. G.; Al-Laham, M. A.; Shirley, W. A.; Mantzaris, J. *J. Chem. Phys*. **1988**, *89*, 2193. b) Petersson, G. A.; Al-Laham, M. A. *J. Chem. Phys*.**1991**, *94*, 6081.

(9) a) Fuentealba, P.; Preuss, H.; Stoll, H.; Szentpály, L. V. *Chem. Phys. Lett*. **1982**, *89*, 418. b) Cao, X. Y. Dolg, M. *J. Chem. Phys*. **2001**, *115*, 7348.

(10) Lu, T.; Chen, F. W. *J. Comput. Chem*. **2012**, *33*, 580.

(11) Han, B.-L.; Liu, Z.; Feng, L.; Wang, Z.; Gupta, R. K.; Aikens, C. M.; Tung, C.-H.; Sun, D. Polymorphism in Atomically Precise Cu_{23} Nanocluster Incorporating Tetrahedral $[Cu_4]^0$ Kernel. *J. Am. Chem. Soc*. **2020**, *142*, 5834−5841.

(12) Su, Y. M.; Wang, Z.; Schein, S.; Tung, C. H.; Sun, D. A Keplerian Ag_{90} Nest of Platonic and Archimedean Polyhedra in Different Symmetry Groups. *Nat. Commun*. **2020**, *11*, 3316.

(13) Lu, C.; Tang, Z. Advanced Inorganic Nanoarchitectures from Oriented Self-Assembly. *Adv. Mater.* **2016**, *28*, 1096–1108.
