# Supplementary material for: Atomically Precise Enantiopure Bimetallic Janus Clusters
Source: ACS Cent Sci. 2022 Sep 7;8(9):1258–64. doi: 10.1021/acscentsci.2c00754 (PMC9523771; doi:10.1021/acscentsci.2c00754)

checkCIF/PLATON report

You have not supplied any structure factors. As a result the full set of tests cannot be run.

THIS REPORT IS FOR GUIDANCE ONLY. IF USED AS PART OF A REVIEW PROCEDURE FOR PUBLICATION, IT SHOULD NOT REPLACE THE EXPERTISE OF AN EXPERIENCED CRYSTALLOGRAPHIC REFEREE.

No syntax errors found. CIF dictionary Interpreting this report

Datablock: rac-cu4au8

```
Bond precision:   C-C = 0.0267 A          Wavelength=1.54184

Cell:              a=17.2705(1)      b=16.4232(1)      c=22.2882(1)
                   alpha=90          beta=99.2500      gamma=90
Temperature:       200 K
```

	Calculated	Reported
Volume	6239.55(6)	6239.57(6)
Space group	P n	P 1 n 1
Hall group	P -2yac	P -2yac
Moiety formula	2(C120 H90 Au8 Cu4 N6 P6 S6), C H2 Cl2 [+ solvent]	C120 H90 Au8 Cu4 N6 P6 S6, 0.5(C H2 Cl2)
Sum formula	C241 H182 Au16 Cl2 Cu8 N12 P12 S12 [+ solvent]	C120.50 H91 Au8 Cl Cu4 N6 P6 S6
Mr	7733.18	3866.51
Dx,g cm-3	2.058	2.058
Z	1	2
Mu (mm-1)	20.141	20.141
F000	3614.0	3614.0
F000’	3542.16	
h,k,lmax	21,20,27	21,20,27
Nref	25400[12710]	19719
Tmin,Tmax	0.122,0.133	0.875,1.000
Tmin’	0.019	

```
Correction method= # Reported T Limits: Tmin=0.875 Tmax=1.000
AbsCorr = MULTI-SCAN

Data completeness= 1.55/0.78        Theta(max)= 74.109
```

R(reflections)= 0.0351(18944)   wR2(reflections)= 0.0911(19719)

S = 1.073   Npar= 1552

The following ALERTS were generated. Each ALERT has the format
test-name_ALERT_alert-type_alert-level.
Click on the hyperlinks for more details of the test.

Alert level B

```
PLAT342_ALERT_3_B Low Bond Precision on  C-C Bonds ...............    0.02674 Ang.
```

Alert level C

```
PLAT220_ALERT_2_C NonSolvent   Resd 1  C   Ueq(max)/Ueq(min) Range        3.8 Ratio
PLAT234_ALERT_4_C Large Hirshfeld Difference P1       --C13      .       0.16 Ang.
PLAT234_ALERT_4_C Large Hirshfeld Difference P1       --C25      .       0.16 Ang.
PLAT234_ALERT_4_C Large Hirshfeld Difference P5       --C91A     .       0.18 Ang.
PLAT234_ALERT_4_C Large Hirshfeld Difference C5       --C6       .       0.22 Ang.
PLAT234_ALERT_4_C Large Hirshfeld Difference C69      --C70      .       0.24 Ang.
PLAT241_ALERT_2_C High   'MainMol' Ueq as Compared to Neighbors of        C22 Check
PLAT241_ALERT_2_C High   'MainMol' Ueq as Compared to Neighbors of        C35 Check
PLAT241_ALERT_2_C High   'MainMol' Ueq as Compared to Neighbors of        C41 Check
PLAT241_ALERT_2_C High   'MainMol' Ueq as Compared to Neighbors of        C63 Check
PLAT241_ALERT_2_C High   'MainMol' Ueq as Compared to Neighbors of        C69 Check
PLAT241_ALERT_2_C High   'MainMol' Ueq as Compared to Neighbors of       C107 Check
PLAT242_ALERT_2_C Low    'MainMol' Ueq as Compared to Neighbors of        C37 Check
PLAT260_ALERT_2_C Large Average Ueq of Residue Including       Cl1      0.143 Check
PLAT721_ALERT_1_C Bond    Calc     1.41(5), Rep     1.39000 Dev...       0.02 Ang.
              C88A     -C87A           1_555   1_555 ........     #  256 Check
```

Alert level G

```
PLAT002_ALERT_2_G Number of Distance or Angle Restraints on AtSite         16 Note
PLAT003_ALERT_2_G Number of Uiso or Uij Restrained non-H Atoms ...         73 Report
PLAT042_ALERT_1_G Calc. and Reported Moiety Formula Strings Differ     Please Check
PLAT045_ALERT_1_G Calculated and Reported Z Differ by a Factor ...     0.5000 Check
PLAT083_ALERT_2_G SHELXL Second Parameter in WGHT  Unusually Large      20.64 Why ?
PLAT142_ALERT_4_G s.u. on b - Axis Small or Missing .............    0.00010 Ang.
PLAT143_ALERT_4_G s.u. on c - Axis Small or Missing .............    0.00010 Ang.
PLAT145_ALERT_4_G s.u. on beta     Small or Missing .............     0.0000 Degree
PLAT172_ALERT_4_G The CIF-Embedded .res File Contains DFIX Records          6 Report
PLAT177_ALERT_4_G The CIF-Embedded .res File Contains DELU Records          2 Report
PLAT186_ALERT_4_G The CIF-Embedded .res File Contains ISOR Records          5 Report
PLAT232_ALERT_2_G Hirshfeld Test Diff (M-X)  Cu2      --S3       .        5.5 s.u.
PLAT300_ALERT_4_G Atom Site Occupancy of Cl1        Constrained at        0.5 Check
PLAT300_ALERT_4_G Atom Site Occupancy of Cl2        Constrained at        0.5 Check
PLAT300_ALERT_4_G Atom Site Occupancy of C0AA       Constrained at        0.5 Check
PLAT300_ALERT_4_G Atom Site Occupancy of H0AA       Constrained at        0.5 Check
PLAT300_ALERT_4_G Atom Site Occupancy of H0AB       Constrained at        0.5 Check
PLAT301_ALERT_3_G Main Residue  Disorder ..............(Resd  1  )        21% Note
PLAT302_ALERT_4_G Anion/Solvent/Minor-Residue Disorder (Resd  2  )       100% Note
PLAT304_ALERT_4_G Non-Integer Number of Atoms in ..... (Resd  2  )       2.50 Check
PLAT432_ALERT_2_G Short Inter X...Y Contact  Cl2      ..C1A      .       3.21 Ang.
                                             1/2+x,-y,1/2+z  =      2_555 Check
```

```
PLAT606_ALERT_4_G Solvent Accessible VOID(S) in Structure ........          ! Info
PLAT720_ALERT_4_G Number of Unusual/Non-Standard Labels ..........          3 Note
PLAT722_ALERT_1_G Angle   Calc      117.00, Rep      118.10 Dev...       1.10 Degree
              C90     -C89     -H89        1_555  1_555  1_555     #  595 Check
PLAT794_ALERT_5_G Tentative Bond Valency for Cu3       (I)       .       1.04 Info
PLAT860_ALERT_3_G Number of Least-Squares Restraints ............         476 Note
PLAT883_ALERT_1_G No Info/Value for _atom_sites_solution_primary .     Please Do !
```

```
   0 ALERT level A = Most likely a serious problem - resolve or explain
   1 ALERT level B = A potentially serious problem, consider carefully
  15 ALERT level C = Check. Ensure it is not caused by an omission or oversight
  27 ALERT level G = General information/check it is not something unexpected

   5 ALERT type 1 CIF construction/syntax error, inconsistent or missing data
  14 ALERT type 2 Indicator that the structure model may be wrong or deficient
   3 ALERT type 3 Indicator that the structure quality may be low
  20 ALERT type 4 Improvement, methodology, query or suggestion
   1 ALERT type 5 Informative message, check
```

It is advisable to attempt to resolve as many as possible of the alerts in all categories. Often the minor alerts point to easily fixed oversights, errors and omissions in your CIF or refinement strategy, so attention to these fine details can be worthwhile. In order to resolve some of the more serious problems it may be necessary to carry out additional measurements or structure refinements. However, the purpose of your study may justify the reported deviations and the more serious of these should normally be commented upon in the discussion or experimental section of a paper or in the "special_details" fields of the CIF. checkCIF was carefully designed to identify outliers and unusual parameters, but every test has its limitations and alerts that are not important in a particular case may appear. Conversely, the absence of alerts does not guarantee there are no aspects of the results needing attention. It is up to the individual to critically assess their own results and, if necessary, seek expert advice.

Publication of your CIF in IUCr journals

A basic structural check has been run on your CIF. These basic checks will be run on all CIFs submitted for publication in IUCr journals (*Acta Crystallographica*, *Journal of Applied Crystallography*, *Journal of Synchrotron Radiation*); however, if you intend to submit to *Acta Crystallographica Section C* or *E* or *IUCrData*, you should make sure that full publication checks are run on the final version of your CIF prior to submission.

Publication of your CIF in other journals

Please refer to the *Notes for Authors* of the relevant journal for any special instructions relating to CIF submission.

PLATON version of 20/01/2022; check.def file version of 19/01/2022

Datablock rac-cu4au8 - ellipsoid plot

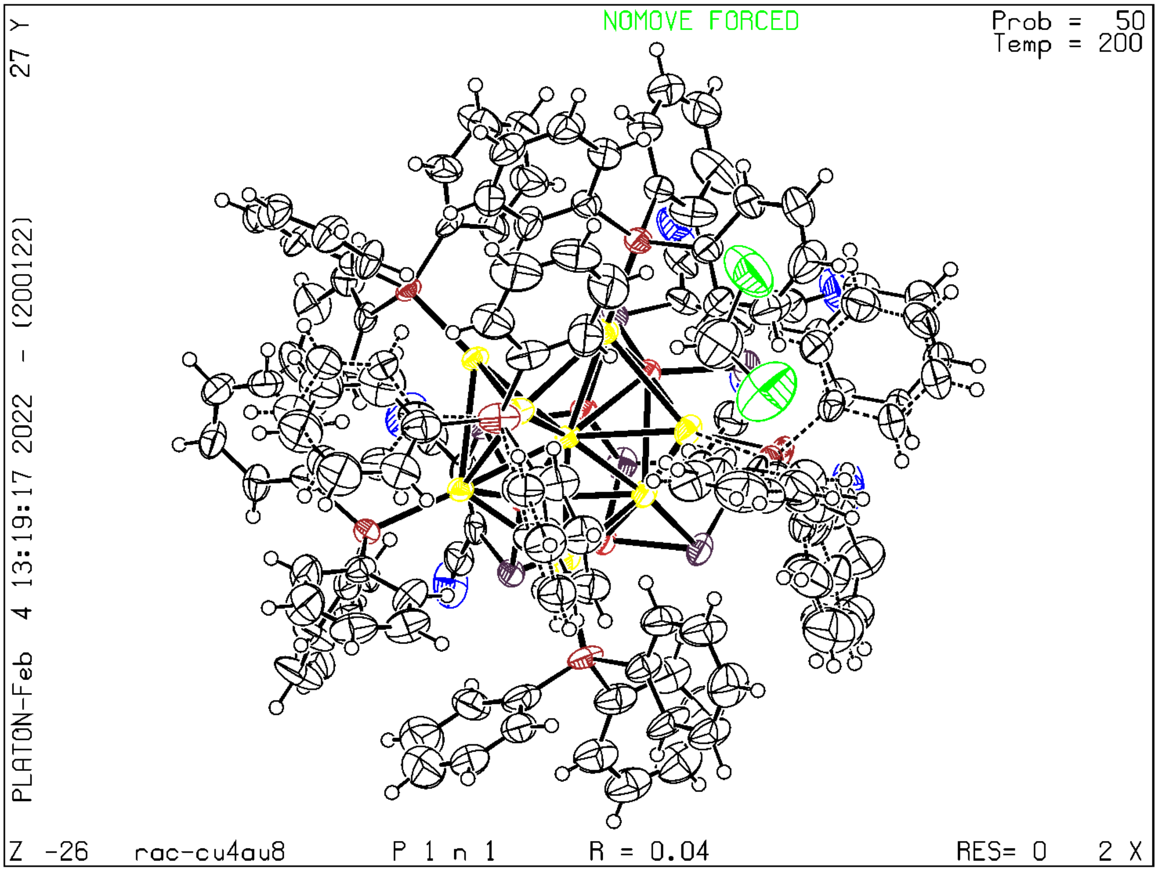

Supplement: Supplementary file 6 — oc2c00754_si_006.pdf [file oc2c00754_si_006.pdf]
