# Supplementary material for: Atomically Precise Enantiopure Bimetallic Janus Clusters
Source: ACS Cent Sci. 2022 Sep 7;8(9):1258–64. doi: 10.1021/acscentsci.2c00754 (PMC9523771; doi:10.1021/acscentsci.2c00754)

checkCIF/PLATON report

Structure factors have been supplied for datablock(s) r-cu4au8

THIS REPORT IS FOR GUIDANCE ONLY. IF USED AS PART OF A REVIEW PROCEDURE FOR PUBLICATION, IT SHOULD NOT REPLACE THE EXPERTISE OF AN EXPERIENCED CRYSTALLOGRAPHIC REFEREE.

No syntax errors found.   CIF dictionary   Interpreting this report

Datablock: r-cu4au8

Bond precision:	C-C = 0.0299 A		Wavelength=1.54184
Cell:	a=16.5742(1)	b=26.8134(1)	c=31.7444(1)
	alpha=90	beta=91.417(1)	gamma=90
Temperature:	100 K		

	Calculated	Reported
Volume	14103.24(11)	14103.24(11)
Space group	P 21	P 1 21 1
Hall group	P 2yb	P 2yb
Moiety formula	2(C144 H96 Au8 Cu4 N6 P6 S6), 3(C H2 Cl2) [+ solvent]	2(C144 H96 Au8 Cu4 N6 P6 S6), 3(C H2 Cl2)
Sum formula	C291 H198 Au16 Cl6 Cu8 N12 P12 S12 [+ solvent]	C291 H198 Au16 Cl6 Cu8 N12 P12 S12
Mr	8491.60	8491.45
Dx,g cm-3	2.000	2.000
Z	2	2
Mu (mm-1)	18.239	18.239
F000	7996.0	7996.0
F000’	7856.67	
h,k,lmax	20,33,39	20,33,39
Nref	57083[29182]	54572
Tmin,Tmax	0.065,0.112	0.259,1.000
Tmin’	0.010	

Correction method= # Reported T Limits: Tmin=0.259 Tmax=1.000
AbsCorr = MULTI-SCAN

Data completeness= 1.87/0.96   Theta(max)= 73.775

R(reflections)= 0.0519(51759)   wR2(reflections)= 0.1416(54572)

S = 1.051   Npar= 3190

The following ALERTS were generated. Each ALERT has the format
test-name_ALERT_alert-type_alert-level.
Click on the hyperlinks for more details of the test.

Alert level B

```
PLAT342_ALERT_3_B Low Bond Precision on  C-C Bonds ...............    0.02986 Ang.
```

Alert level C

```
PLAT213_ALERT_2_C Atom C8              has ADP max/min Ratio .....        3.1 oblate
PLAT213_ALERT_2_C Atom P8              has ADP max/min Ratio .....        3.3 oblate
PLAT213_ALERT_2_C Atom C185            has ADP max/min Ratio .....        3.6 oblate
PLAT220_ALERT_2_C NonSolvent   Resd 1  C   Ueq(max)/Ueq(min) Range        3.3 Ratio
PLAT220_ALERT_2_C NonSolvent   Resd 2  C   Ueq(max)/Ueq(min) Range        3.6 Ratio
PLAT241_ALERT_2_C High   'MainMol' Ueq as Compared to Neighbors of        C23 Check
PLAT241_ALERT_2_C High   'MainMol' Ueq as Compared to Neighbors of       C217 Check
PLAT241_ALERT_2_C High   'MainMol' Ueq as Compared to Neighbors of       C237 Check
PLAT241_ALERT_2_C High   'MainMol' Ueq as Compared to Neighbors of       C239 Check
PLAT242_ALERT_2_C Low    'MainMol' Ueq as Compared to Neighbors of         C1 Check
PLAT242_ALERT_2_C Low    'MainMol' Ueq as Compared to Neighbors of       C238 Check
PLAT244_ALERT_4_C Low    'Solvent' Ueq as Compared to Neighbors of       C1AA Check
PLAT244_ALERT_4_C Low    'Solvent' Ueq as Compared to Neighbors of       C0A0 Check
PLAT260_ALERT_2_C Large Average Ueq of Residue Including      Cl1      0.157 Check
PLAT260_ALERT_2_C Large Average Ueq of Residue Including      Cl01     0.120 Check
PLAT260_ALERT_2_C Large Average Ueq of Residue Including      Cl0A     0.134 Check
PLAT331_ALERT_2_C Small Aver Phenyl C-C Dist C163     --C168      .     1.37 Ang.
PLAT332_ALERT_2_C Large Phenyl C-C Range     C083      -C205      .     0.18 Ang.
PLAT334_ALERT_2_C Small Aver. Benzene C-C Dist C28      -C33            1.37 Ang.
PLAT369_ALERT_2_C Long   C(sp2)-C(sp2) Bond  C78      - C79       .     1.53 Ang.
PLAT790_ALERT_4_C Centre of Gravity not Within Unit Cell: Resd.  #         1 Note
              C144 H96 Au8 Cu4 N6 P6 S6
PLAT911_ALERT_3_C Missing FCF Refl Between Thmin & STh/L=    0.600        91 Report
PLAT977_ALERT_2_C Check Negative Difference Density on H0AD       .    -0.36 eA-3
PLAT977_ALERT_2_C Check Negative Difference Density on H18        .    -0.53 eA-3
PLAT977_ALERT_2_C Check Negative Difference Density on H87        .    -0.38 eA-3
PLAT977_ALERT_2_C Check Negative Difference Density on H89        .    -0.37 eA-3
PLAT977_ALERT_2_C Check Negative Difference Density on H103       .    -0.54 eA-3
PLAT977_ALERT_2_C Check Negative Difference Density on H108       .    -0.37 eA-3
PLAT977_ALERT_2_C Check Negative Difference Density on H118       .    -0.48 eA-3
PLAT977_ALERT_2_C Check Negative Difference Density on H211       .    -0.36 eA-3
PLAT977_ALERT_2_C Check Negative Difference Density on H258       .    -0.32 eA-3
PLAT977_ALERT_2_C Check Negative Difference Density on H269       .    -0.31 eA-3
PLAT977_ALERT_2_C Check Negative Difference Density on H286       .    -0.42 eA-3
```

Alert level G

```
PLAT002_ALERT_2_G Number of Distance or Angle Restraints on AtSite         6 Note
PLAT003_ALERT_2_G Number of Uiso or Uij Restrained non-H Atoms ...        47 Report
PLAT083_ALERT_2_G SHELXL Second Parameter in WGHT  Unusually Large     62.12 Why ?
PLAT142_ALERT_4_G s.u. on b - Axis Small or Missing .............    0.00010 Ang.
```

```
PLAT143_ALERT_4_G s.u. on c - Axis Small or Missing .............    0.00010 Ang.
PLAT153_ALERT_1_G The s.u.'s on the Cell Axes   are Equal ..(Note)     0.0001 Ang.
PLAT172_ALERT_4_G The CIF-Embedded .res File Contains DFIX Records          1 Report
PLAT177_ALERT_4_G The CIF-Embedded .res File Contains DELU Records          2 Report
PLAT178_ALERT_4_G The CIF-Embedded .res File Contains SIMU Records          2 Report
PLAT186_ALERT_4_G The CIF-Embedded .res File Contains ISOR Records         14 Report
PLAT333_ALERT_2_G Large Aver C6-Ring C-C Dist C212     -C221     .       1.43 Ang.
PLAT333_ALERT_2_G Large Aver C6-Ring C-C Dist C222     -C231     .       1.42 Ang.
PLAT333_ALERT_2_G Large Aver C6-Ring C-C Dist C256     -C265     .       1.42 Ang.
PLAT606_ALERT_4_G Solvent Accessible VOID(S) in Structure ........          ! Info
PLAT720_ALERT_4_G Number of Unusual/Non-Standard Labels ..........         13 Note
PLAT790_ALERT_4_G Centre of Gravity not Within Unit Cell: Resd.  #          2 Note
              C144 H96 Au8 Cu4 N6 P6 S6
PLAT790_ALERT_4_G Centre of Gravity not Within Unit Cell: Resd.  #          4 Note
              C H2 Cl2
PLAT791_ALERT_4_G Model has Chirality at P1          (Sohnke SpGr)          R Verify
PLAT791_ALERT_4_G Model has Chirality at P4          (Sohnke SpGr)          R Verify
PLAT794_ALERT_5_G Tentative Bond Valency for Cu3       (I)       .       1.02 Info
PLAT860_ALERT_3_G Number of Least-Squares Restraints .............        319 Note
PLAT883_ALERT_1_G No Info/Value for _atom_sites_solution_primary .     Please Do !
PLAT910_ALERT_3_G Missing # of FCF Reflection(s) Below Theta(Min).          3 Note
PLAT912_ALERT_4_G Missing # of FCF Reflections Above STh/L=  0.600        425 Note
PLAT933_ALERT_2_G Number of HKL-OMIT Records in Embedded .res File         21 Note
PLAT978_ALERT_2_G Number C-C Bonds with Positive Residual Density.          0 Info
```

---

0 **ALERT level A** = Most likely a serious problem - resolve or explain
1 **ALERT level B** = A potentially serious problem, consider carefully
33 **ALERT level C** = Check. Ensure it is not caused by an omission or oversight
26 **ALERT level G** = General information/check it is not something unexpected

2 ALERT type 1 CIF construction/syntax error, inconsistent or missing data
37 ALERT type 2 Indicator that the structure model may be wrong or deficient
4 ALERT type 3 Indicator that the structure quality may be low
16 ALERT type 4 Improvement, methodology, query or suggestion
1 ALERT type 5 Informative message, check

It is advisable to attempt to resolve as many as possible of the alerts in all categories. Often the minor alerts point to easily fixed oversights, errors and omissions in your CIF or refinement strategy, so attention to these fine details can be worthwhile. In order to resolve some of the more serious problems it may be necessary to carry out additional measurements or structure refinements. However, the purpose of your study may justify the reported deviations and the more serious of these should normally be commented upon in the discussion or experimental section of a paper or in the "special_details" fields of the CIF. checkCIF was carefully designed to identify outliers and unusual parameters, but every test has its limitations and alerts that are not important in a particular case may appear. Conversely, the absence of alerts does not guarantee there are no aspects of the results needing attention. It is up to the individual to critically assess their own results and, if necessary, seek expert advice.

Publication of your CIF in IUCr journals

A basic structural check has been run on your CIF. These basic checks will be run on all CIFs submitted for publication in IUCr journals (*Acta Crystallographica*, *Journal of Applied Crystallography*, *Journal of Synchrotron Radiation*); however, if you intend to submit to *Acta Crystallographica Section C* or *E* or *IUCrData*, you should make sure that full publication checks are run on the final version of your CIF prior to submission.

Publication of your CIF in other journals

Please refer to the *Notes for Authors* of the relevant journal for any special instructions relating to CIF submission.

PLATON version of 20/01/2022; check.def file version of 19/01/2022

Datablock r-cu4au8 - ellipsoid plot

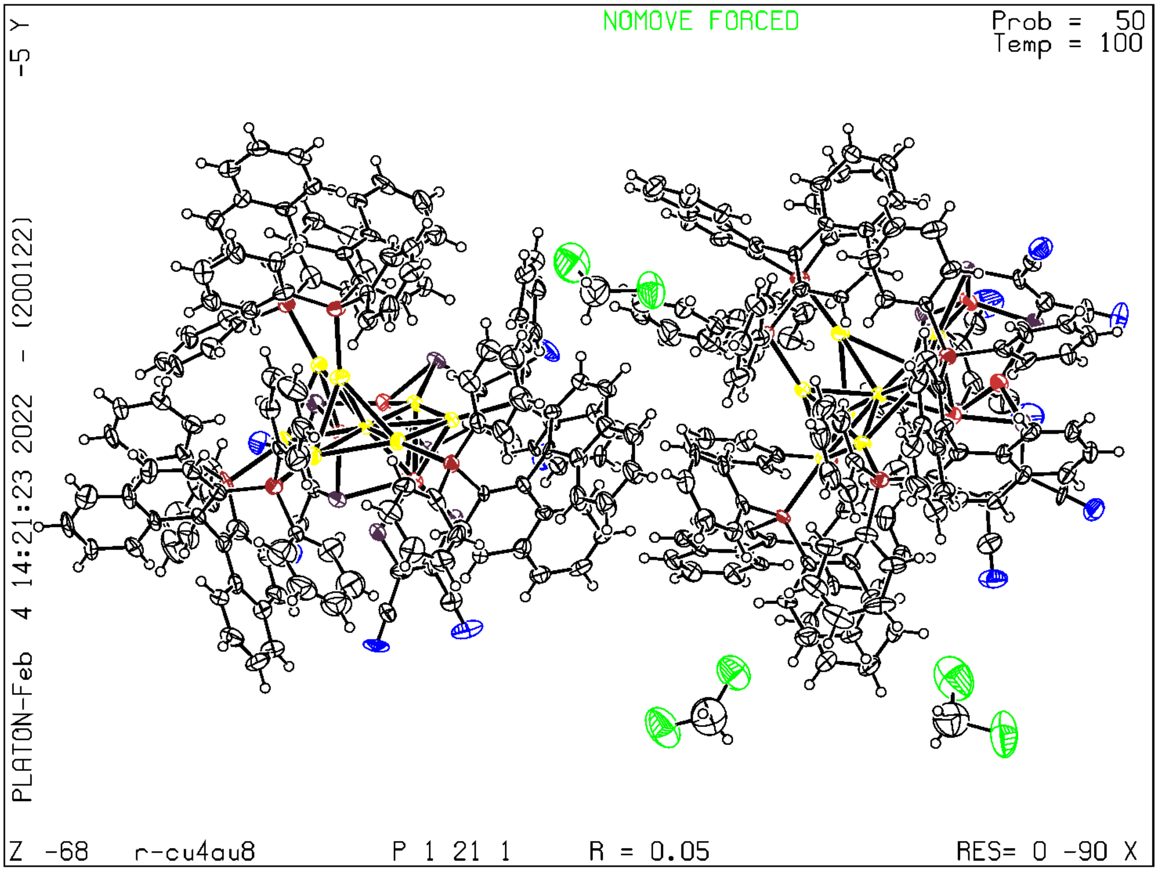

Supplement: Supplementary file 7 — oc2c00754_si_007.pdf [file oc2c00754_si_007.pdf]
