# Supplementary material for: Atomically Precise Enantiopure Bimetallic Janus Clusters
Source: ACS Cent Sci. 2022 Sep 7;8(9):1258–64. doi: 10.1021/acscentsci.2c00754 (PMC9523771; doi:10.1021/acscentsci.2c00754)

# checkCIF/PLATON report

Structure factors have been supplied for datablock(s) s-cu4au8

THIS REPORT IS FOR GUIDANCE ONLY. IF USED AS PART OF A REVIEW PROCEDURE FOR PUBLICATION, IT SHOULD NOT REPLACE THE EXPERTISE OF AN EXPERIENCED CRYSTALLOGRAPHIC REFEREE.

No syntax errors found. CIF dictionary Interpreting this report

## Datablock: s-cu4au8

---

| | | | |
|---|---|---|---|
| Bond precision: | C-C = 0.0580 A | | Wavelength=1.54184 |
| Cell: | a=16.6336(2) | b=26.8950(3) | c=31.7629(4) |
| | alpha=90 | beta=91.346(1) | gamma=90 |
| Temperature: | 200 K | | |

| | Calculated | Reported |
|---|---|---|
| Volume | 14205.6(3) | 14205.6(3) |
| Space group | P 21 | P 1 21 1 |
| Hall group | P 2yb | P 2yb |
| Moiety formula | C144 H96 Au8 Cu4 N6 P6 S6 [+ solvent] | C144 H96 Au8 Cu4 N6 P6 S6 |
| Sum formula | C144 H96 Au8 Cu4 N6 P6 S6 [+ solvent] | C144 H96 Au8 Cu4 N6 P6 S6 |
| Mr | 4118.41 | 4118.33 |
| Dx,g cm-3 | 1.926 | 1.926 |
| Z | 4 | 4 |
| Mu (mm-1) | 17.577 | 17.577 |
| F000 | 7744.0 | 7744.0 |
| F000’ | 7600.06 | |
| h,k,lmax | 20,33,39 | 20,33,39 |
| Nref | 57792[ 29541] | 53322 |
| Tmin,Tmax | 0.119,0.121 | 0.531,1.000 |
| Tmin’ | 0.020 | |

Correction method= # Reported T Limits: Tmin=0.531 Tmax=1.000
AbsCorr = MULTI-SCAN

Data completeness= 1.81/0.92 Theta(max)= 74.127

R(reflections)= 0.0808( 42412)

wR2(reflections)= 0.2239( 53322)

S = 1.043 Npar= 3121

---

The following ALERTS were generated. Each ALERT has the format
**test-name_ALERT_alert-type_alert-level.**
Click on the hyperlinks for more details of the test.

---

## Alert level B

```
PLAT342_ALERT_3_B Low Bond Precision on  C-C Bonds ..............      0.058 Ang.
```

---

## Alert level C

```
PLAT094_ALERT_2_C Ratio of Maximum / Minimum Residual Density ....       2.28 Report
PLAT213_ALERT_2_C Atom C222            has ADP max/min Ratio .....        3.9 prolat
PLAT220_ALERT_2_C NonSolvent   Resd 1  C   Ueq(max)/Ueq(min) Range        3.8 Ratio
PLAT220_ALERT_2_C NonSolvent   Resd 2  C   Ueq(max)/Ueq(min) Range        3.1 Ratio
PLAT241_ALERT_2_C High   'MainMol' Ueq as Compared to Neighbors of        C31 Check
PLAT241_ALERT_2_C High   'MainMol' Ueq as Compared to Neighbors of        C54 Check
PLAT241_ALERT_2_C High   'MainMol' Ueq as Compared to Neighbors of        C61 Check
PLAT241_ALERT_2_C High   'MainMol' Ueq as Compared to Neighbors of        C86 Check
PLAT241_ALERT_2_C High   'MainMol' Ueq as Compared to Neighbors of        C88 Check
PLAT241_ALERT_2_C High   'MainMol' Ueq as Compared to Neighbors of       C102 Check
PLAT241_ALERT_2_C High   'MainMol' Ueq as Compared to Neighbors of       C118 Check
PLAT241_ALERT_2_C High   'MainMol' Ueq as Compared to Neighbors of       C141 Check
PLAT241_ALERT_2_C High   'MainMol' Ueq as Compared to Neighbors of       C161 Check
PLAT241_ALERT_2_C High   'MainMol' Ueq as Compared to Neighbors of       C167 Check
PLAT241_ALERT_2_C High   'MainMol' Ueq as Compared to Neighbors of       C207 Check
PLAT241_ALERT_2_C High   'MainMol' Ueq as Compared to Neighbors of       C210 Check
PLAT241_ALERT_2_C High   'MainMol' Ueq as Compared to Neighbors of       C215 Check
PLAT241_ALERT_2_C High   'MainMol' Ueq as Compared to Neighbors of       C240 Check
PLAT241_ALERT_2_C High   'MainMol' Ueq as Compared to Neighbors of       C241 Check
PLAT241_ALERT_2_C High   'MainMol' Ueq as Compared to Neighbors of       C247 Check
PLAT241_ALERT_2_C High   'MainMol' Ueq as Compared to Neighbors of       C249 Check
PLAT241_ALERT_2_C High   'MainMol' Ueq as Compared to Neighbors of       C254 Check
PLAT241_ALERT_2_C High   'MainMol' Ueq as Compared to Neighbors of       C287 Check
PLAT242_ALERT_2_C Low    'MainMol' Ueq as Compared to Neighbors of         C1 Check
PLAT242_ALERT_2_C Low    'MainMol' Ueq as Compared to Neighbors of         C3 Check
PLAT242_ALERT_2_C Low    'MainMol' Ueq as Compared to Neighbors of       C152 Check
PLAT242_ALERT_2_C Low    'MainMol' Ueq as Compared to Neighbors of       C162 Check
PLAT242_ALERT_2_C Low    'MainMol' Ueq as Compared to Neighbors of       C208 Check
PLAT242_ALERT_2_C Low    'MainMol' Ueq as Compared to Neighbors of       C211 Check
PLAT242_ALERT_2_C Low    'MainMol' Ueq as Compared to Neighbors of       C221 Check
PLAT242_ALERT_2_C Low    'MainMol' Ueq as Compared to Neighbors of       C239 Check
PLAT242_ALERT_2_C Low    'MainMol' Ueq as Compared to Neighbors of       C246 Check
PLAT331_ALERT_2_C Small Aver Phenyl C-C Dist C201     --C206     .       1.37 Ang.
PLAT332_ALERT_2_C Large Phenyl C-C Range     C163      -C168     .       0.16 Ang.
PLAT332_ALERT_2_C Large Phenyl C-C Range     C189      -C194     .       0.18 Ang.
PLAT332_ALERT_2_C Large Phenyl C-C Range     C251      -C256     .       0.17 Ang.
PLAT911_ALERT_3_C Missing FCF Refl Between Thmin & STh/L=    0.600         51 Report
PLAT977_ALERT_2_C Check Negative Difference Density on H22       .      -0.37 eA-3
PLAT977_ALERT_2_C Check Negative Difference Density on H27       .      -0.33 eA-3
PLAT977_ALERT_2_C Check Negative Difference Density on H30       .      -0.45 eA-3
PLAT977_ALERT_2_C Check Negative Difference Density on H37       .      -0.34 eA-3
```

```
PLAT977_ALERT_2_C Check Negative Difference Density on H64       .      -0.43 eA-3
PLAT977_ALERT_2_C Check Negative Difference Density on H66       .      -0.48 eA-3
PLAT977_ALERT_2_C Check Negative Difference Density on H74       .      -0.31 eA-3
PLAT977_ALERT_2_C Check Negative Difference Density on H83       .      -0.48 eA-3
PLAT977_ALERT_2_C Check Negative Difference Density on H84       .      -0.41 eA-3
PLAT977_ALERT_2_C Check Negative Difference Density on H89       .      -0.38 eA-3
PLAT977_ALERT_2_C Check Negative Difference Density on H111      .      -0.31 eA-3
PLAT977_ALERT_2_C Check Negative Difference Density on H131      .      -0.44 eA-3
PLAT977_ALERT_2_C Check Negative Difference Density on H158      .      -0.49 eA-3
PLAT977_ALERT_2_C Check Negative Difference Density on H161      .      -0.40 eA-3
PLAT977_ALERT_2_C Check Negative Difference Density on H170      .      -0.59 eA-3
PLAT977_ALERT_2_C Check Negative Difference Density on H175      .      -0.32 eA-3
PLAT977_ALERT_2_C Check Negative Difference Density on H176      .      -0.33 eA-3
PLAT977_ALERT_2_C Check Negative Difference Density on H186      .      -0.47 eA-3
PLAT977_ALERT_2_C Check Negative Difference Density on H187      .      -0.43 eA-3
PLAT977_ALERT_2_C Check Negative Difference Density on H210      .      -0.57 eA-3
PLAT977_ALERT_2_C Check Negative Difference Density on H211      .      -0.32 eA-3
PLAT977_ALERT_2_C Check Negative Difference Density on H220      .      -0.34 eA-3
PLAT977_ALERT_2_C Check Negative Difference Density on H225      .      -0.33 eA-3
PLAT977_ALERT_2_C Check Negative Difference Density on H240      .      -0.63 eA-3
PLAT977_ALERT_2_C Check Negative Difference Density on H259      .      -0.33 eA-3
PLAT977_ALERT_2_C Check Negative Difference Density on H271      .      -0.61 eA-3
PLAT977_ALERT_2_C Check Negative Difference Density on H286      .      -0.34 eA-3
```

---

### Alert level G

```
PLAT002_ALERT_2_G Number of Distance or Angle Restraints on AtSite         52 Note
PLAT003_ALERT_2_G Number of Uiso or Uij Restrained non-H Atoms ...         98 Report
PLAT072_ALERT_2_G SHELXL First  Parameter in WGHT  Unusually Large       0.13 Report
PLAT083_ALERT_2_G SHELXL Second Parameter in WGHT  Unusually Large     148.53 Why ?
PLAT172_ALERT_4_G The CIF-Embedded .res File Contains DFIX Records         11 Report
PLAT177_ALERT_4_G The CIF-Embedded .res File Contains DELU Records          1 Report
PLAT186_ALERT_4_G The CIF-Embedded .res File Contains ISOR Records         16 Report
PLAT335_ALERT_2_G Check Large C6 Ring C-C Range C69      -C78            0.16 Ang.
PLAT335_ALERT_2_G Check Large C6 Ring C-C Range C79      -C88            0.16 Ang.
PLAT335_ALERT_2_G Check Large C6 Ring C-C Range C267     -C276           0.18 Ang.
PLAT606_ALERT_4_G Solvent Accessible VOID(S) in Structure ........          ! Info
PLAT722_ALERT_1_G Angle   Calc      121.00, Rep      119.80 Dev...       1.20 Degree
              C199    -C198    -H198       1_555  1_555  1_555     #  930 Check
PLAT722_ALERT_1_G Angle   Calc      117.00, Rep      118.40 Dev...       1.40 Degree
              C256    -C255    -H255       1_555  1_555  1_555     #  961 Check
PLAT722_ALERT_1_G Angle   Calc      123.00, Rep      124.10 Dev...       1.10 Degree
              C144    -C143    -H143       1_555  1_555  1_555     # 1275 Check
PLAT722_ALERT_1_G Angle   Calc      120.00, Rep      118.70 Dev...       1.30 Degree
              C137    -C136    -H136       1_555  1_555  1_555     # 1288 Check
PLAT794_ALERT_5_G Tentative Bond Valency for Cu3       (I)       .       1.00 Info
PLAT860_ALERT_3_G Number of Least-Squares Restraints ............         651 Note
PLAT883_ALERT_1_G No Info/Value for _atom_sites_solution_primary .     Please Do !
PLAT910_ALERT_3_G Missing # of FCF Reflection(s) Below Theta(Min).          2 Note
PLAT912_ALERT_4_G Missing # of FCF Reflections Above STh/L=  0.600        593 Note
PLAT933_ALERT_2_G Number of HKL-OMIT Records in Embedded .res File          9 Note
PLAT941_ALERT_3_G Average HKL Measurement Multiplicity ..........         4.3 Low
PLAT978_ALERT_2_G Number C-C Bonds with Positive Residual Density.          0 Info
PLAT992_ALERT_5_G Repd & Actual _reflns_number_gt Values Differ by          3 Check
```

---

0 **ALERT level A** = Most likely a serious problem - resolve or explain

```
 1 ALERT level B = A potentially serious problem, consider carefully
64 ALERT level C = Check. Ensure it is not caused by an omission or oversight
24 ALERT level G = General information/check it is not something unexpected

 5 ALERT type 1 CIF construction/syntax error, inconsistent or missing data
72 ALERT type 2 Indicator that the structure model may be wrong or deficient
 5 ALERT type 3 Indicator that the structure quality may be low
 5 ALERT type 4 Improvement, methodology, query or suggestion
 2 ALERT type 5 Informative message, check
```

---

It is advisable to attempt to resolve as many as possible of the alerts in all categories. Often the minor alerts point to easily fixed oversights, errors and omissions in your CIF or refinement strategy, so attention to these fine details can be worthwhile. In order to resolve some of the more serious problems it may be necessary to carry out additional measurements or structure refinements. However, the purpose of your study may justify the reported deviations and the more serious of these should normally be commented upon in the discussion or experimental section of a paper or in the "special_details" fields of the CIF. checkCIF was carefully designed to identify outliers and unusual parameters, but every test has its limitations and alerts that are not important in a particular case may appear. Conversely, the absence of alerts does not guarantee there are no aspects of the results needing attention. It is up to the individual to critically assess their own results and, if necessary, seek expert advice.

**Publication of your CIF in IUCr journals**

A basic structural check has been run on your CIF. These basic checks will be run on all CIFs submitted for publication in IUCr journals (*Acta Crystallographica*, *Journal of Applied Crystallography*, *Journal of Synchrotron Radiation*); however, if you intend to submit to *Acta Crystallographica Section C* or *E* or *IUCrData*, you should make sure that full publication checks are run on the final version of your CIF prior to submission.

**Publication of your CIF in other journals**

Please refer to the *Notes for Authors* of the relevant journal for any special instructions relating to CIF submission.

---

**PLATON version of 20/01/2022; check.def file version of 19/01/2022**

**Datablock s-cu4au8** - ellipsoid plot

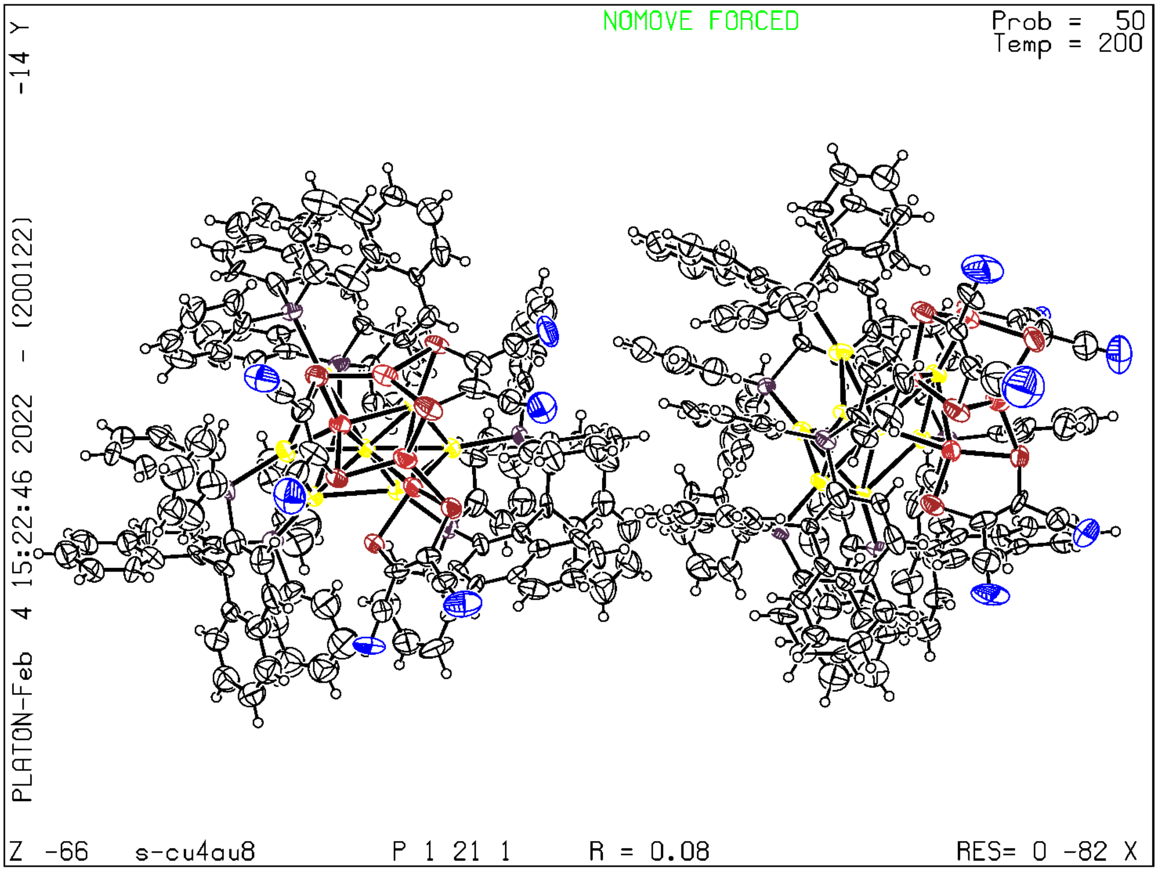

Supplement: Supplementary file 8 — oc2c00754_si_008.pdf [file oc2c00754_si_008.pdf]
