# Supplementary material for: Atomically Precise Enantiopure Bimetallic Janus Clusters
Source: ACS Cent Sci. 2022 Sep 7;8(9):1258–64. doi: 10.1021/acscentsci.2c00754 (PMC9523771; doi:10.1021/acscentsci.2c00754)

checkCIF/PLATON report

You have not supplied any structure factors. As a result the full set of tests cannot be run.

THIS REPORT IS FOR GUIDANCE ONLY. IF USED AS PART OF A REVIEW PROCEDURE FOR PUBLICATION, IT SHOULD NOT REPLACE THE EXPERTISE OF AN EXPERIENCED CRYSTALLOGRAPHIC REFEREE.

No syntax errors found. CIF dictionary Interpreting this report

Datablock: au3cd

```
Bond precision:   C-C = 0.0164 A          Wavelength=1.54184

Cell:              a=22.9702(2)      b=23.8042(2)      c=27.0023(3)
                   alpha=90          beta=90           gamma=90
Temperature:       200 K
```

	Calculated	Reported
Volume	14764.5(2)	14764.5(2)
Space group	P b c a	P b c a
Hall group	-P 2ac 2ab	-P 2ac 2ab
Moiety formula	C79 H66 Au3 Cd Cl N2 P6 S2, C H4 O	C79 H66 Au3 Cd Cl N2 P6 S2, C H4 O
Sum formula	C80 H70 Au3 Cd Cl N2 O P6 S2	C80 H70 Au3 Cd Cl N2 O P6 S2
Mr	2064.09	2064.07
Dx,g cm-3	1.857	1.857
Z	8	8
Mu (mm-1)	15.689	15.689
F000	7968.0	7968.0
F000'	7900.17	
h,k,lmax	28,29,33	28,29,32
Nref	15073	14686
Tmin,Tmax	0.108,0.095	0.091,1.000
Tmin'	0.017	

```
Correction method= # Reported T Limits: Tmin=0.091 Tmax=1.000
AbsCorr = MULTI-SCAN

Data completeness= 0.974            Theta(max)= 74.384
```

R(reflections)= 0.0544(11698) wR2(reflections)= 0.1551(14686)

S = 1.029 Npar= 959

The following ALERTS were generated. Each ALERT has the format
test-name_ALERT_alert-type_alert-level.
Click on the hyperlinks for more details of the test.

Alert level C

```
PLAT094_ALERT_2_C Ratio of Maximum / Minimum Residual Density ....       2.22 Report
PLAT234_ALERT_4_C Large Hirshfeld Difference C20      --C21      .       0.22 Ang.
PLAT241_ALERT_2_C High   'MainMol' Ueq as Compared to Neighbors of         S2 Check
PLAT241_ALERT_2_C High   'MainMol' Ueq as Compared to Neighbors of        C59 Check
PLAT241_ALERT_2_C High   'MainMol' Ueq as Compared to Neighbors of        C69 Check
PLAT241_ALERT_2_C High   'MainMol' Ueq as Compared to Neighbors of        C72 Check
PLAT241_ALERT_2_C High   'MainMol' Ueq as Compared to Neighbors of        C73 Check
PLAT242_ALERT_2_C Low    'MainMol' Ueq as Compared to Neighbors of         C5 Check
PLAT242_ALERT_2_C Low    'MainMol' Ueq as Compared to Neighbors of        C68 Check
PLAT242_ALERT_2_C Low    'MainMol' Ueq as Compared to Neighbors of        C71 Check
PLAT260_ALERT_2_C Large Average Ueq of Residue Including        O1      0.148 Check
PLAT342_ALERT_3_C Low Bond Precision on  C-C Bonds ...............    0.01637 Ang.
```

Alert level G

```
PLAT002_ALERT_2_G Number of Distance or Angle Restraints on AtSite         22 Note
PLAT003_ALERT_2_G Number of Uiso or Uij Restrained non-H Atoms ...         29 Report
PLAT007_ALERT_5_G Number of Unrefined Donor-H Atoms .............          1 Report
PLAT083_ALERT_2_G SHELXL Second Parameter in WGHT  Unusually Large      94.44 Why ?
PLAT142_ALERT_4_G s.u. on b - Axis Small or Missing .............    0.00020 Ang.
PLAT172_ALERT_4_G The CIF-Embedded .res File Contains DFIX Records          4 Report
PLAT173_ALERT_4_G The CIF-Embedded .res File Contains DANG Records          1 Report
PLAT174_ALERT_4_G The CIF-Embedded .res File Contains FLAT Records          3 Report
PLAT177_ALERT_4_G The CIF-Embedded .res File Contains DELU Records          1 Report
PLAT186_ALERT_4_G The CIF-Embedded .res File Contains ISOR Records          6 Report
PLAT301_ALERT_3_G Main Residue  Disorder .............(Resd  1  )         11% Note
PLAT793_ALERT_4_G Model has Chirality at P4          (Centro SPGR)          S Verify
PLAT860_ALERT_3_G Number of Least-Squares Restraints .............        214 Note
PLAT883_ALERT_1_G No Info/Value for _atom_sites_solution_primary .     Please Do !
PLAT933_ALERT_2_G Number of HKL-OMIT Records in Embedded .res File          1 Note
PLAT941_ALERT_3_G Average HKL Measurement Multiplicity ...........        3.7 Low
```

0 **ALERT level A** = Most likely a serious problem - resolve or explain
0 **ALERT level B** = A potentially serious problem, consider carefully
12 **ALERT level C** = Check. Ensure it is not caused by an omission or oversight
16 **ALERT level G** = General information/check it is not something unexpected

1 ALERT type 1 CIF construction/syntax error, inconsistent or missing data
14 ALERT type 2 Indicator that the structure model may be wrong or deficient
4 ALERT type 3 Indicator that the structure quality may be low
8 ALERT type 4 Improvement, methodology, query or suggestion
1 ALERT type 5 Informative message, check

It is advisable to attempt to resolve as many as possible of the alerts in all categories. Often the minor alerts point to easily fixed oversights, errors and omissions in your CIF or refinement strategy, so attention to these fine details can be worthwhile. In order to resolve some of the more serious problems it may be necessary to carry out additional measurements or structure refinements. However, the purpose of your study may justify the reported deviations and the more serious of these should normally be commented upon in the discussion or experimental section of a paper or in the "special_details" fields of the CIF. checkCIF was carefully designed to identify outliers and unusual parameters, but every test has its limitations and alerts that are not important in a particular case may appear. Conversely, the absence of alerts does not guarantee there are no aspects of the results needing attention. It is up to the individual to critically assess their own results and, if necessary, seek expert advice.

Publication of your CIF in IUCr journals

A basic structural check has been run on your CIF. These basic checks will be run on all CIFs submitted for publication in IUCr journals (*Acta Crystallographica*, *Journal of Applied Crystallography*, *Journal of Synchrotron Radiation*); however, if you intend to submit to *Acta Crystallographica Section C* or *E* or *IUCrData*, you should make sure that full publication checks are run on the final version of your CIF prior to submission.

Publication of your CIF in other journals

Please refer to the *Notes for Authors* of the relevant journal for any special instructions relating to CIF submission.

PLATON version of 20/01/2022; check.def file version of 19/01/2022

Datablock au3cd - ellipsoid plot

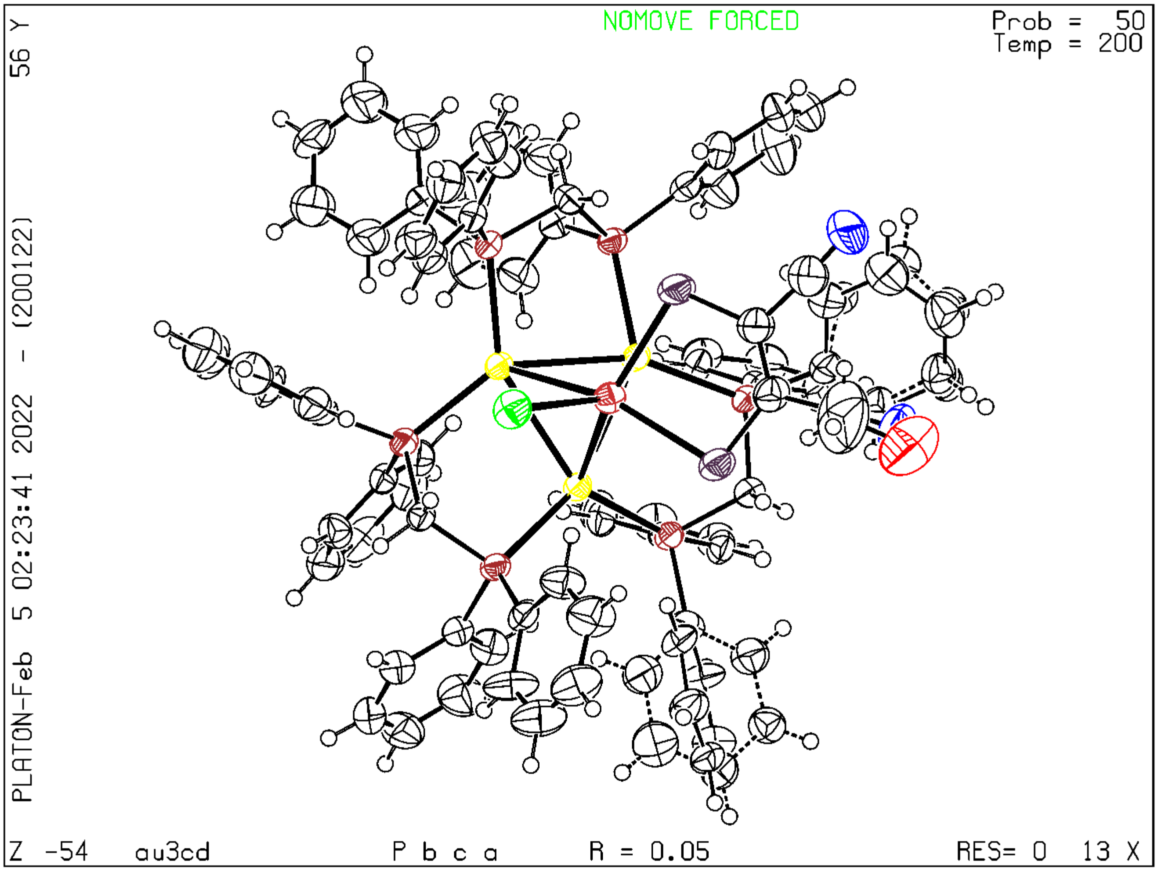

Supplement: Supplementary file 9 — oc2c00754_si_009.pdf [file oc2c00754_si_009.pdf]
